# Synthesis, Molecular Docking, and Biofilm Formation Inhibitory Activity of Bis(Indolyl)Pyridines Analogues of the Marine Alkaloid Nortopsentin

**DOI:** 10.3390/molecules26144112

**Published:** 2021-07-06

**Authors:** Heba M. Abo-Salem, Hayam A. Abd El Salam, Anhar M. Abdel-Aziem, Mohamed S. Abdel-Aziz, Eslam Reda El-Sawy

**Affiliations:** 1 Chemistry of Natural Compounds Department, National Research Centre, Dokki, Giza 12622, Egypt; hb_abosalem@yahoo.com; 2 Green Chemistry Department, National Research Centre, Dokki, Giza 12622, Egypt; hayam_nrc@yahoo.com; 3 Chemistry Department, Faculty of Science (Girl’s Branch), Al-Azhar University, Cairo 11284, Egypt; anharabdelaziem@gmail.com; 4 Microbial Chemistry Department, Genetic Engineering and biotechnology Division, National Research Centre, Dokki, Giza 12622, Egypt; mohabomerna@yahoo.ca

**Keywords:** nortopsentin, bis(indolyl)pyridine, benzofuran, antimicrobial, biofilm formation, molecular docking

## Abstract

An efficient and simple protocol for the synthesis of a new class of diverse bis(indolyl)pyridines analogues of the marine alkaloid nortopsentin has been reported. A one-pot four-component condensation of 3-cyanocarbomethylindole, various aldehyde, 3-acetylindole, and ammonium acetate in glacial acetic acid led to the formation of 2,6-bis(1*H*-indol-3-yl)-4-(substituted-phenyl)pyridine-5-carbonitriles. Additionally, 2,6-bis(1*H*-indol-3-yl)-4-(benzofuran) pyridine-5-carbonitriles were prepared via a one-pot four-component condensation of 3-cyanocarbomethylindole, various *N*-substituted-indole-3-aldehydes, 2-acetylbenzofuran, and ammonium acetate. The synthesized compounds were evaluated for their ability to inhibit biofilm formation against the Gram-positive bacterial reference strains *Staphylococcus aureus* ATCC 6538 and the Gram-negative strain *Escherichia coli* ATCC 25922. Some of the new compounds showed a marked selectivity against the Gram-positive and Gram-negative strains. Remarkably, five compounds **4b**, **7a**, **7c**, **7d** and **8e** demonstrated good antibiofilm formation against *S. aureus* and *E. coli*. On the other hand, the release of reducing sugars and proteins from the treated bacterial strains over the untreated strains was considered to explain the disruption effect of the selected compound on the contact cells of *S. aureus* and *E. coli*. Out of all studied compounds, the binding energies and binding mode of bis-indole derivatives **7c** and **7d** were theoretically the best thymidylate kinase, DNA gyrase B and DNA topoisomerase IV subunit B inhibitors.

## 1. Introduction

The recent high challenges in our health system are to overcome the increment of pathogen antibiotic-resistant microbes. These multidrug-resistant (MDR) strains are the reasons for serious public health problems [1]. The common drug-resistant Gram-positive bacteria include *Staphylococcus aureus*, *Enterococcus faecium*, *Enterococcus faecalis* as well as *Streptococcus pneumoniae* whereas the common drug-resistant Gram-negative bacteria are *Escherichia coli*, *Klebsiella pneumoniae*, *Pseudomonas aeruginosa* and *Actinobacter baumannii* [2]. Production of degrading enzymes, low permeability of the bacterial outer membranes, efflux pumps, and modification of targets are patterns of mechanisms used by bacteria to resist the toxicity of antibiotics [3]. About 60–80% of bacterial infections are biofilm-mediated [4].

Microbial biofilm is the major reason for the failure of any antibiotic to kill microbial pathogens [5,6]. Biofilm is known as a community of microbes fixed in self-produced polymeric materials cemented to living or non-living surfaces [5,6]. These polymeric materials include exopolysaccharides, extracellular DNA, proteins and amyloidogenic proteins [5,6]. 

Recently, many efforts have been made to evaluate several compounds as antibiofilm agents [7,8], till now, no derivative has reached clinical use. Therefore, there is a vital necessity for the development of new small molecules able to be effective in inhibiting biofilm formation or in dispersing preformed biofilm [6].

**Figure 1:**
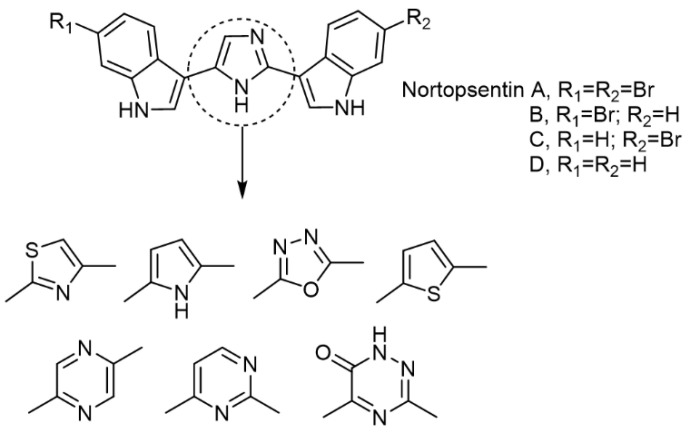
The structure of nortopsentins A–D and their related analogues.

Nortopsentins A–D, one of the bis(indolyl)alkaloids with a characteristic 2,4-bis(3ʹ-indolyl)imidazole skeleton and were isolated from the sponge *Spongosorites ruetzler* [9,10]. Nortopsentins A-C exhibited in vitro cytotoxicity against P388 leukemia cells, additionally, they display antifungal activity against *Candida albicans* [11]. On the other hand, nortopsentin A inhibited parasite growth at the trophozoite stage at submicromolar inhibitory concentrations [12]. Since nortopsentins have versatile applications, research trials of different structures of the bis-indole-based scaffold were attempted, by replacing the imidazole ring with other heterocycles. Wide varieties of heterocycles including thiazole [6,13], isoxazole [14], furan [14], thiophene [15], pyrazole [16], pyrrole [17], pyrazine [18], triazinone [19] rings were constructed by a synthetic pathway to introduced various analogues of the marine nortopsentins (Figure 1). 

Several studies proven that, the analogues of the marine nortopsentins showed diverse biological activities *viz* antitumor [16,19,20,21], CDK1 inhibitors [22,23], antiviral [24], antifungal [24], insecticidal agents [11,25]. Recently published work on the analogues of the marine nortopsentins has focused on the development of synthetic analogs as inhibitors of bacterial biofilm formation [6,13]. They have proven that thiazole nortopsentin analogues showed a typical anti-virulence profile, being able to inhibit the biofilm formation without affecting the microbial growth in the planktonic form [6,13]. On the other hand, the pyridine nucleus was shown to be effective as it inhibits bacterial biofilm formation [26,27].

Based on the aforementioned attributes, our study aimed to obtain potent anti-biofilm agents that could effectively treat Gram-positive bacterial reference *strains Staphylococcus aureus* and the Gram-negative strain *Escherichia coli* that are biofilm-mediated. In the present study, we report the synthesis of a new series of bis(indolyl)pyridine derivatives, structurally related to the nortopsentin and evaluate their ability to inhibit biofilm formation. Additionally, to investigate the mechanism of the most active antimicrobial compounds, molecular docking studies were carried out on three bacterial target enzymes; thymidylate kinase, DNA gyrase B, and DNA topoisomerase IV subunit B.

## 2. Results and Discussion

### 2.1. Chemistry

**Scheme 1:**
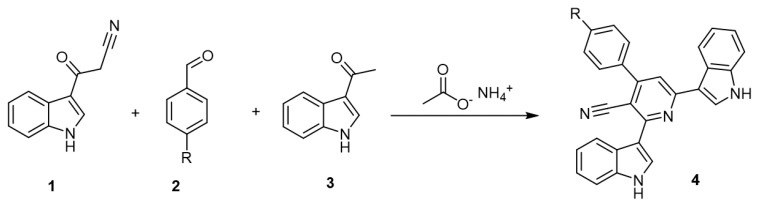
A one-pot four-component condensation reaction to synthesize 2,6-bis(1*H*-indol-3-yl)-4-(substituted-phenyl)pyridin-5-carbonitriles; **2a**–**j** and **4a**–**j**, a, R=H; b, R=Br; c, R=Cl; d, R=F; e, R=OCH_3_; f, R=3,5-(OCH_3_)_2_; g, R=3,4,5-(OCH_3_)_3_; h, R=NO_2_; i, R=N,N-(CH_3_)_2_; j, R=OH. Reagents and conditions: glacial acetic acid, ammonium acetate (10 mmol), reflux, 2 h.

**Scheme 2:**
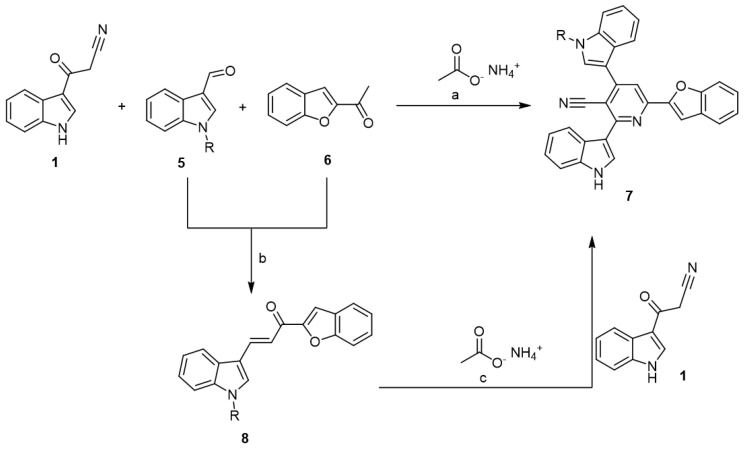
A one-pot four-component condensation reaction for the synthesis of 2,6-bis(1*H*-indol-3-yl) -4-(benzofuran-2-yl)pyridin-5-carbonitriles; **5a**–**e**, **7a**–**e**, and **8a**–**e**; a, R=H; b, R=CH_2_CH_3_; c, R= CH_2_Ph; d, R=COPh; e, R=SO_2_Ph. Reagents and conditions. (a) glacial acetic acid, ammonium acetate (10 mmol), reflux, 2–3 h; (b) EtOH, piperidine, reflux; (c) glacial acetic acid, ammonium acetate (10 mmol), reflux, 7–10 h.

Up to our knowledge, Jiang and his coworkers are the only authors who described the synthesis of bis(indolyl)pyridines via the Suzuki cross-coupling reaction between 2,6-dichloro-4-trifluoromethylpyridine and *N*-tosyl-3-indolylboronic acid [28]. In the present work, we introduce a new class of bis-indole containing pyridine-3-carbonitrile as nortopsentin analogues via a simple and convenient synthetic pathway as outlined in Scheme 1 and Scheme 2.

First, we reported the synthesis of bis(indolyl)pyridine derivatives incorporated with a simple substituted phenyl. The starting 3-(1*H*-indol-3-yl)-3-oxopropanenitrile (**1**) was prepared via the electrophilic substitution reaction of indole with cyanoacetic acid [29]. Condensation of 3-(1*H*-indol-3-yl)-3-oxopropanenitrile (**1**) with various substituted aldehydes **2**, 3-indolyl methyl ketone (**3**), and ammonium acetate under reflux in glacial acetic acid following a one-pot four-component domino protocol led to the formation of 2,6-bis(1*H*-indol-3-yl)-4-(substituted-phenyl)pyridin-5-carbonitriles **4a**–**j** (Scheme 1). 

We observed that the amount of ammonium acetate affects the reaction yield. Where, using two equivalents of ammonium acetate was unsuccessful to give the preferred product yield even after a long reaction time of 24 h, especially with the phenyl ring substituted with a donating group (OH, NR_2_). Then, the model reaction was attempted by exceeding the amount of ammonium acetate 10-folds resulting in a good yield (60–96%). Among all of them, the 2,6-di(1*H*-indol-3-yl)-4-(4-methoxyphenyl)pyridine-3-carbonitrile (**4e**) was found to be superior with a product yield of 96%.

Next, a new series of bis-indole-pyridine-benzofuran, namely 6-(benzofuran-2-yl)-4-(1-substituted-1*H*-indol-3-yl)-2-(1*H*-indol-3-yl)pyridine-3-carbonitriles **7a**–**e** was rationally designed and synthesized (Scheme 2). Due to various pharmacological features of benzofuran derivatives [30,31,32], we were encouraged to initiate further reaction studies by replacing the substituted phenyl of compounds **4a**–**j** with benzofuran. Thus, *N*-substituted-1*H*-indole-3-carboxaldhydes **5a**–**e** was achieved to react with 3-(1*H*-indol-3-yl)-3-oxopropanenitrile (**1**), followed by the addition of 2-benzofuranyl methyl ketone (**6**) and ammonium acetate under reflux in glacial acetic acid under a one-pot four-component domino protocol (Scheme 2). 

**Scheme 3:**
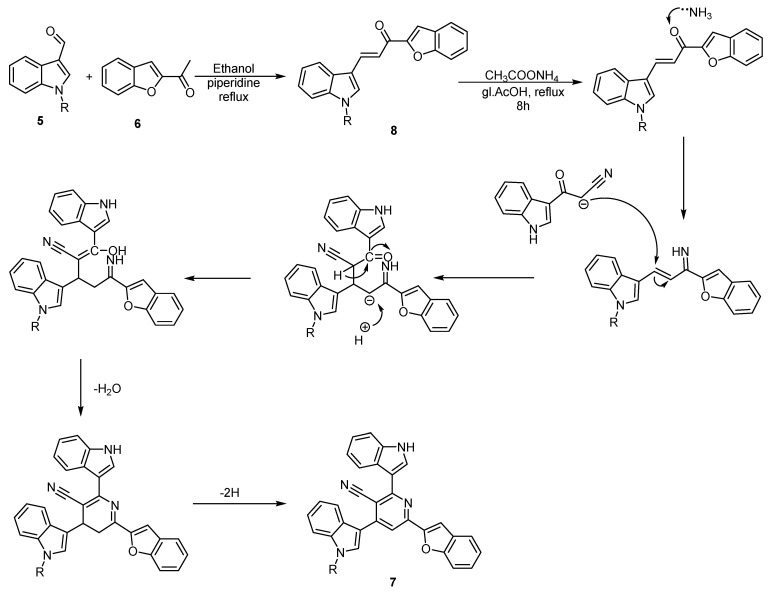
Synthetic pathway of the *α,β*-unsaturated ketones **8** in the formation of 2,6-bis(1*H*-indol-3-yl)-4-(benzofuran-2-yl)pyridin-5-carbonitriles **7**.

To widen our research, the *α,β*-unsaturated carbonyl systems, namely (*E*)-1-(benzofuran-2-yl)-3-(*N*-substituted-indol-3-yl)prop-2-en-1-ones **8a**–**e** were prepared as valuable and versatile intermediates for the synthesis of bis(indolyl)pyridines **7a**–**e** (Scheme 2). Thus, Claisen-Schmidt condensation of *N*-substituted-1*H*-indole-3-carboxaldhydes **5a**–**e** with 2-benzofuranyl methyl ketone (**6**) in presence of piperidine as a base under heating afforded the pure products of *α,β*-unsaturated ketones **8a**–**e** in good yield 74–85%. The resulting *α,β*-unsaturated ketones **8a**–**e** were converted into the corresponding bis(indolyl)pyridines **7a**–**e** in excellent yields via their reaction with 3-(1*H*-indol-3-yl)-3-oxopropanenitrile (**1**) and ammonium acetate under heating in glacial acetic acid (Scheme 2). The yields obtained for compounds **7a**–**e** through the two pathways were not much different and the yields were good in most cases from 50 to 85%. The plausible mechanism for compounds **7a**–**e** via the cyclization of *α,β*-unsaturated ketones **8a**–**e** was described in Scheme 3. 

### 2.2. Biological Studies

**Figure 2:**
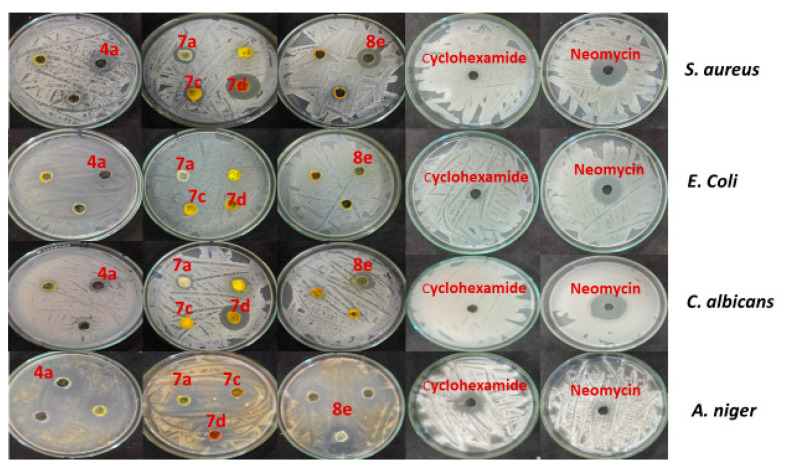
The inhibition zone (фmm) of the most active selected compounds against various pathogenic microorganisms.

All synthesized compounds were preliminary tested in vitro antimicrobial activity against a variety of pathogenic microorganisms, namely *Staphylococcus aureus* ATCC 6538 (Gram-positive bacteria), *Escherichia coli* ATCC 25922 (Gram-negative bacteria), *Candida albicans* ATCC 10231 (yeast), and *Aspergillus niger* NRRL A-326 (fungus) using the cup plate diffusion method at a single dose of 250 μg/100 μL. The results are shown in (Table 1 and Figure 2) as the growth inhibition zone (mm). It was found that compounds **4b**, **7a**, **7c**, **7d,** and **8e** exhibited relatively high antimicrobial activities against most tested microbes. Where they revealed inhibition values of 20, 18, 17, 36, and 21 mm against *S. aureus*. Furthermore, they showed inhibition values of 15, 0, 13, 12, and 14 mm against *E. coli*. In the case of *C. albicans* the inhibition values were 16, 17, 12, 27, and 22 mm. The inhibition values were 26, 13, 0, 14, and 14 mm for *A. niger*. The other compounds showed relatively moderate to low antimicrobial activity against most tested microbes.

For more explanation, the structure–activity relationship, (SAR) investigations demonstrated that the antibacterial activity of the target compounds is influenced by the activity of substituents. In the case of halogens as electron-withdrawing substituents, weak activity is observed when chlorine and fluorine atoms are substituted to the benzene ring while the presence of a bromine atom enhances the activity. Accordingly, their antimicrobial activity was in the increasing order of **4b** (Br) > **4c** (Cl) > **4d** (F). On the other hand, the presence of *p*-methoxy phenyl as an electron-donating substituent decreases the biological activity in order of **4e** (OCH_3_) > **4f** (3,5-(OCH_3_)_2_) > **4g** (3,4,5-(OCH_3_)_3_). The enhanced activity of compound 7b could be explained by the presence of the benzoyl group.

Further work was performed to determine the minimum inhibitory concentrations (MICs), and the minimum bactericidal concentrations (MBCs) values of the most active selected compounds **4b**, **7a**, **7c**, **7d**, and **8e**. It was found from Table 2 that, compounds **7d** and **8e** revealed the lowest MIC and MBC values of 9.766, 19.53 and 19.53, 19.53 μg/mL against *S. aureus*, and 19.53, 19.53 and 19.53, 39.06 μg/mL against *C. albicans*. Considerably lower MIC and MBC values were noticed with compounds **4b** and **7a** with values of 78.125, 312.5, and 39.063, 625 μg/mL, respectively for *S. aureus*. Regarding *C. albicans* the MIC and MBC values of **4b** and **7a** were 78, 312.5, and 39.06, 156.25 μg/mL, respectively. Compound **7c** exhibited higher MIC and MBC values against all tested microbes except *C. albicans* with values of 39.06, and 78 μg/mL. All compounds exhibited higher MIC and MBC values with *E. coli* (Table 2). 

### 2.3. Inhibition of Biofilm Formation

**Figure 3:**
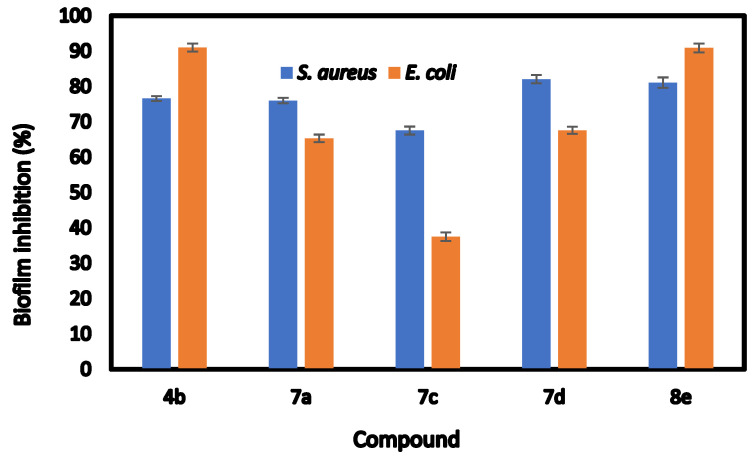
Antibiofilm formation (inhibition %) of the most active selected compounds against *Staphylococcus aureus* (blue), and *Escherichia coli* (orange). Values are expressed as means ± SE; *n* = 3 for each group.

**Figure 4:**
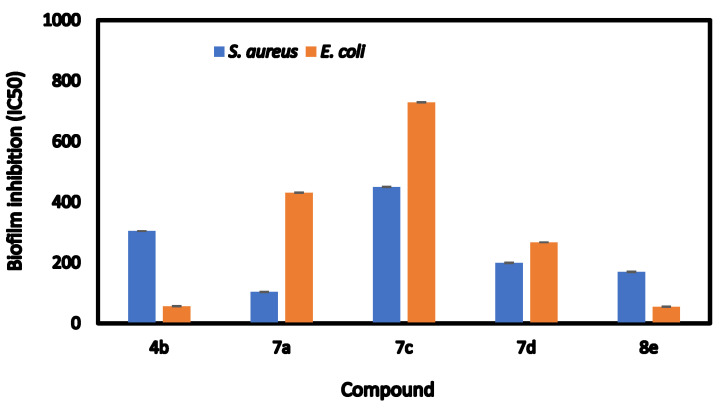
Antibiofilm formation (IC_50_, μg/mL) of the most active selected compounds against *Staphylococcus aureus* (blue), and *Escherichia coli* (orange). Values are expressed as means ± SE; *n* = 3 for each group.

Microorganisms with the ability to produce biofilms are known as one of the major factors contributing to antibiotic resistance. Therefore, many trials were established to overcome these severe problems by searching for new drugs that could inhibit biofilm formation [4]. Table 3, Figure 3 and Figure 4 explained the ability of the most active selected compounds as antibiofilm formation. It was found that compounds **4b**, **7a**, **7d** and **8e** exhibited considerably good antibiofilm formation with inhibition values of 76.59, 76.00, 82.06, and 81.08%, respectively, against *S. aureus*, whereas compound **7c** revealed lower inhibition values of 67.52% against the same test microbe. For *E. coli*, compounds **4b** and **8e** showed higher biofilm inhibition values of 91.02, and 90.88%, respectively. On the other hand, compounds **7a** and **7d** had moderate antibiofilm against *E. coli* with values of 65.33, and 67.60%, respectively. Additionally, compound **7c** showed low biofilm inhibition of 37.5%.

### 2.4. Release of Reducing Sugars and Proteins

**Figure 5:**
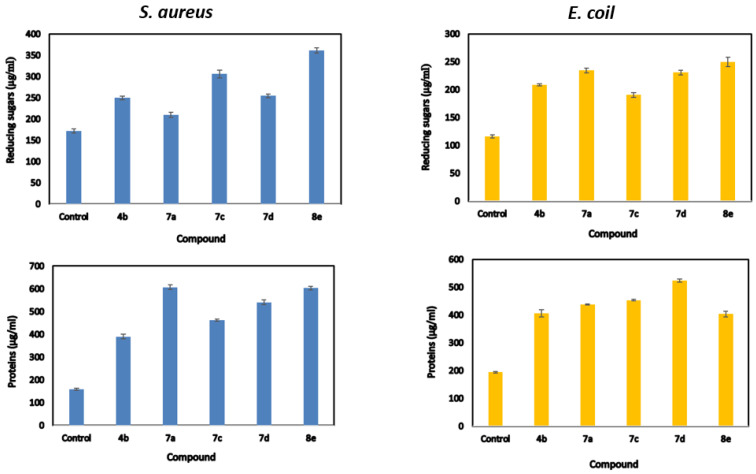
Reducing sugars and proteins released from treated Gram-positive (*Staphylococcus aureus*) in blue color, and Gram-negative bacteria (*Escherichia coli*) in yellow color with the most active selected compounds compared to untreated cultures (control).

The leakage of biomolecules such as reducing sugars and proteins was considered as a tool explaining the effect of active compounds on test microbes [33]. All compounds exhibited release of reducing sugars and proteins more than controls (*S. aureus* and *E. coli*) with variable values (Table 4, Figure 5). Some antibiotics could inhibit the biosynthesis of cell wall peptidoglycan and their cell become more susceptible to osmotic lysis and thus become toxic to the bacterial cell, as human cells do not have peptidoglycan.

### 2.5. Molecular Docking Results

From the literature, nortopsentin analogues have antimicrobial activity and inhibit bacterial biofilm formation but their mechanism of action has not been studied yet [6]. Therefore, we need to choose three of the most important antimicrobial target enzymes to study the ability of our compounds to inhibit them using molecular docking technique; the best methods to expect the inhibitory activity theoretically. In addition, the presence of the pyridine ring encourages us to study the tree enzymes, since pyridine has a broad spectrum of biological activity and is a good inhibitor for Gyrase B and topoisomerase [34].

The proliferation of antibiotic-resistant strains of pathogenic bacteria has led to a decrease in antibiotics’ usefulness in the clinic [35]. 

To avoid this cross-resistance, new agents must be developed that can act either through novel mechanisms of action or against unique binding sites on existing validated targets. Target selection is critical to decreasing the chance of developing resistance [36]. 

Compounds capable of interacting with multiple sites on the same target or with different enzymes were shown to reduce the ability of bacteria to develop resistance [37].

From the above point of view, the mechanisms of action of the most active compounds **4b**, **7a**, **7c**, **7d,** and **8e** were investigated against different bacterial enzymes using the MOE program in comparison to their native ligands. These mechanisms include: a) thymidylate kinase (PDB ID: 4QGG), a potential therapeutic target for the development of new antibacterial medicines because it is a key enzyme in bacterial DNA reproduction [38]; b) Gyrase B (PDB ID: 6F86), and topoisomerase IV subunit B (PDB ID: 4HZ5) were considered important bacterial enzymes that regulate the topological state of DNA during replication [37].

**Figure 6:**
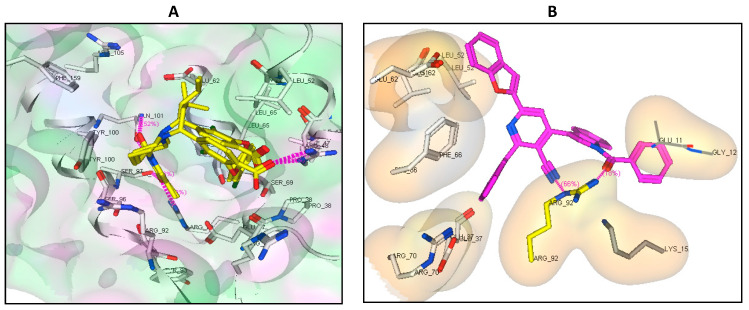
(**A**) The binding mode of the re-docked ligand within the binding pocket of TMPK (ID: 4QGG). The re-docked ligand shows superimposed on the same position as the native ligand (yellow, stick) with the same orientation. The two ligands show the same H-bonds profile with Gln101, Arg48, Arg70, Ser97 (white, stick); (**B**) The binding mode of compound **7d** (pink, stick) within the binding pocket of TMPK (ID: 4QGG). Compound **7d** shows π-cation interaction and two H-bound acceptors with the amine acid residue Arg92 (yellow, stick).

**Figure 7:**
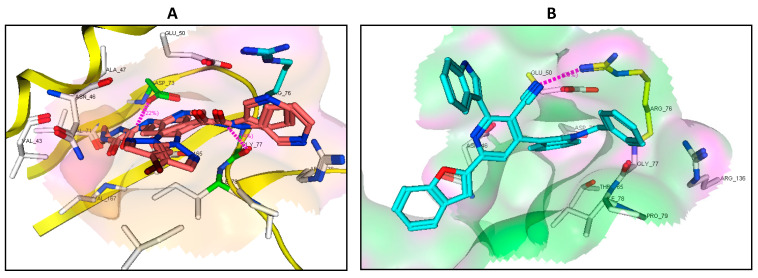
(**A**) The binding mode of the re-docked ligand within the binding pocket of DNA gyrase B (ID: **6F86**). The re-docked ligand shows superimposed on the same position as the native ligand (brown, stick) with the same orientation. The two ligands show the same H-bonds profile with Asp73 and Gly77 (green, stick). The re-docked ligand shows hydrophobic interaction with Arg76 (cyan, stick); (**B**) The binding mode of compound **7c** (cyan, stick) within the binding pocket of DNA gyrase B (ID: **6F86**) with one H-bond acceptor to Arg76 (yellow, stick).

**Figure 8:**
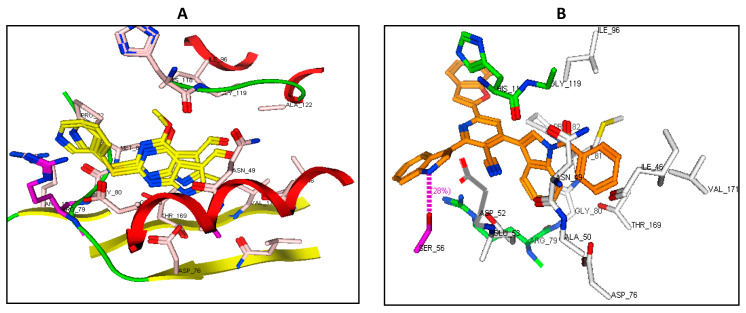
(**A**) The binding mode of the re-docked ligand within the binding pocket of DNA topoisomerase IV subunit B (ID: **4HZ5**). The re-docked ligand shows superimposed on the same position as the native ligand (yellow, stick) with the same orientation. The two ligands show π-cation interaction with Arg79 (pink, stick); (**B**) The binding mode of compound **7d** (brown, stick) within the binding pocket of DNA topoisomerase IV subunit B (ID: **4HZ5**). Compound **7d** shows one H-bond donner with Ser56 (pink, stick) and two π-cation interactions with His118 and Arg79 (green, stick).

The binding mode of the re-docked ligands within the binding pocket of target enzymes showed superimposed on the same position as the native ligands with the same orientation (Figure 6, Figure 7 and Figure 8). Out of all tested compounds, the binding energies of bis-indole derivatives **7c** and **7d** were the best with the three bacterial enzymes (Table 5).

### 2.6. Binding Mode of the Studied Compounds with Thymidylate Kinase (ID: 4QGG)

All antimicrobial active compounds that studied (**4b**, **7a**, **7c**, **7d**, and **8e**) revealed favorable binding interaction and binding energy with the binding site of thymidylate kinase (ID: **4QGG**) with values ranging from −15.52 to −25.36 kcal/mol comparable to the native ligand of −24.34 kcal/mol. 

Compound **7d** revealed the best binding energy of −25.36 kcal/mol, besides good binding interaction with the binding site via formation of π-cation interaction and two H-bound acceptors with the amine acid residue Arg92 (Figure 6).

### 2.7. Binding Mode of the Studied Compounds with DNA Gyrase B (ID: 6F86)

From the docking result, it was observed that only compounds **7c** showed the best binding energy of −22.31 kcal/mol compared to the native ligand of −22.84 kcal/mol (Table 5). Compound **7c** formed one H-bond acceptor with the key amino acids of the ATP binding site Arg76 in a similar pattern as a native ligand (Figure 7).

### 2.8. Binding Mode of the Studied Compounds with DNA Topoisomerase IV Subunit B (ID: 4HZ5)

All the antimicrobial active compounds that were studied revealed better binding energy ranging from −19.09 to −26.53 kcal/mol in comparison to the native ligand of −14.90 kcal/mol (Table 5). In addition, the binding mode of the five selected compounds was similar or better than the native ligand (Table 5). Compounds **7c** and **7d** were theoretically the best DNA topoisomerase IV subunit B inhibitors with a binding energy of −24.90 and −26.53 kcal/mol, respectively, than the native ligand and the rest of the compounds. The binding mode of compound **7d** showed one H-bond donner with the amino acid Ser56, beside π-cation interaction with the amino acid residue His118, and Arg79 in a similar pattern as the native ligand (Figure 8).

## 3. Materials and Methods 

### 3.1. Chemistry 

General Information: All reagents and solvents were of commercial grade. Indole (Sigma-Aldrich Chemie GmeH, Taufkirchen, Germany). Melting points were determined on the digital melting point apparatus (Electro thermal 9100, Electro thermal Engineering Ltd., serial No. 8694, Rochford, United Kingdom) and are uncorrected. The reaction progress was monitored by thin-layer chromatography (TLC) using silica gel plates (POLYGRAM SILG/UV254, 0.20 mm), which were visualized under UV light 254 and 365 nm. Elemental analyses were carried out on a Perkin–Elmer 2400 analyzer (USA) and were found within ± 0.4% of the theoretical values. The ^1^H and ^13^C NMR spectra were measured with a Bruker Avance spectrometer (Bruker, Germany) at 500 and 125 MHz, respectively, using TMS as the internal standard and are available in the Supplementary Materials. Hydrogen coupling patterns are described as (s) singlet, (d) doublet, (t) triplet, (q) quartet and (m) multiple. Chemical shifts were defined as parts per million (ppm) relative to the solvent peak.

*General Procedure for the preparation of 3-(1H-indol-3-yl)-3-oxopropanenitrile (**1**):* A solution of cyanoacetic acid (10 mmol) in acetic anhydride (10 mL) was heated at 50–85 °C for 10 min. Then (10 mmol) of indole was added to the reaction mixture and the heating was continued under reflux for a further 30 min. The formed precipitate on hot was filtered off, dried as a pure compound; colorless crystals with MP 224 –6 °C (reported mp. 224 °C) [29], yield 65%. **^1^H NMR** (500 MHz, DMSO-d_6_) δ= 12.15 (s, NH), 8.33 (s, 1H), 8.10 (d, J = 5 Hz, 1H), 7.48 (d, J = 10 Hz, 1H), 7.22–7.19 (t, J = 15 Hz, 2H), 4.42 (s, CH_2_). 

*General procedure for the preparation of 2,6-Bis(1H-indol-3-yl)-4-(substituted-phenyl)pyridin-5-carbonitriles **4a**–**j**:* A mixture of 3-(1H-indol-3-yl)-3-oxopropanenitrile (**1**, 1.0 mmol) and the appropriate aldehydes (**2**, 1.0 mmol) in glacial acetic acid (20 mL) was stirred at 120 °C for 1 h. Then, 3-indolyl methyl ketone (**3**, 1.0 mmol) and ammonium acetate (10.0 mmol) were added to the reaction mixture and stirred at 120 °C for 2 h. After the reaction was completed (as monitored by TLC), the reaction mixture was cooled to room temperature followed by quenching with ice water. The resulting solid product was filtered off, air-dried and subsequently recrystallized from the proper solvent (obtain *pure* product with column chromatography) to give the pure products **4**. 

*4-Phenyl-2,6-bis(1H-indol-3-yl)pyridine-3-carbonitrile (**4a**):* Recrystallized from methanol as yellow crystals; **MP.** 220–222 °C; **yield**: 0.24 g, 60%; **^1^H NMR** (500 MHz, DMSO-*d*_6_) δ 12.28 (s, 2H, 2NH), 8.44 (s, 2H), 8.21–8.19 (m, 3H), 8.03–8.01 (m, 1H), 7.57–7.53 (m, 6H), 7.27–7.25 (m, 4H); **^13^C NMR** (125 MHz, DMSO-*d*_6_) δ 181.3, 152.0, 136.7, 136.0, 132.4, 132.2, 130.2, 129.1, 126.1, 123.5, 122.4, 121.3, 117.6 (CN), 113.5, 112.5, 111.5; **Anal**. Calcd for C_28_H_18_N_4_ (410.48): C, 81.87; H, 4.37; N, 13.55; found: C, 81.93; H, 4.42; N, 13.65

*4-(4-Bromo-phenyl)-2,6-bis(1H-indol-3-yl)pyridine-3-carbonitrile (**4b**):* Recrystallized from methanol as yellow crystals; **MP.** 188–190 °C; **yield**: 0.29 g, 60%; **^1^H NMR** (500 MHz, DMSO-*d*_6_) δ 12.30 (s, 2H, 2NH), 8.44 (s, 2H), 8.20–8.16 (m, 3H), 8.00–7.79 (m, 3H), 7.82–7.75 (m, 2H), 7.42–7.52 (m, 2H), 7.26–7.24 (m, 3H); **^13^C NMR** (125 MHz, DMSO-*d*_6_) δ 181.7, 151.3, 137.2, 136.8, 132.7, 132.5, 132.3, 126.6, 126.4, 124.1, 123.0, 121.8, 117.9 (CN), 114.0, 113.0, 112.7; **Anal**. Calcd for C_28_H_17_BrN_4_ (489.38): C, 68.72; H, 3.50; Br, 16.33; N, 11.45; found: C, 68.67; H, 3.43; Br, 16.21; N, 11.55.

*4-(4-Chloro-phenyl)-2,6-bis(1H-indol-3-yl)pyridine-3-carbonitrile (**4c**):* Recrystallized from methanol as yellow crystals; **MP.** 197–199 °C; **yield**: 0.33 g, 75%; **^1^H NMR** (500 MHz, DMSO-*d*_6_) δ 12.29 (s, 2H, 2NH), 8.44 (s, 2H), 8.20–8.02 (m, 6H), 7.63–7.53 (m, 5H), 7.32–7.25 (m, 2H); **^13^C NMR** (125 MHz, DMSO-*d*_6_) δ 181.6, 151.1, 137.3, 137.2, 136.7, 132.4, 131.8, 129.7, 126.6, 124.1, 123.0, 121.9, 117.9 (CN), 114.0, 113.0, 112.6; **Anal**. Calcd for C_28_H_17_ClN_4_ (444.92): C, 75.59; H, 3.85; Cl, 7.97; N, 12.59; found: C, 75.61; H, 3.67; Cl, 8.01; N, 13.03.

*4-(4-Fluoro-phenyl)-2,6-bis(1H-indol-3-yl)pyridine-3-carbonitrile (**4d**):* Recrystallized from methanol as yellow crystals; **MP.** 176–178 °C; **yield**: 0.25 g, 60%; **^1^H NMR** (500 MHz, DMSO-*d*_6_) δ 12.29 (s, 2H, 2NH), 8.44 (s, 2H), 8.22–8.01 (m, 5H), 7.52–7.50 (d, *J* = 10 Hz, 2H), 7.42–7.40 (m, 3H), 7.30–7.19 (m, 3H); **^13^C NMR** (125 MHz, DMSO-*d*_6_) δ 181.8, 165.6, 163.5, 151.3, 137.2, 136.6, 133.6, 133.5, 129.6, 126.7, 126.6, 124.1, 122.9, 121.9, 118.1, 116.9 (CN), 116.8, 114.0, 113.0, 111.7; **Anal**. Calcd for C_28_H_17_FN_4_ (428.47): C, 78.49; H, 4.00; F, 4.43; N, 13.08; found: C, 78.33; H, 3.97; F, 4.23; N, 12.98.

*4-(4-Methoxy-phenyl)-2,6-bis(1H-indol-3-yl)pyridine-3-carbonitrile (**4e**).* Recrystallized from methanol as yellow crystals; **MP.** 170–172 °C; **yield**: 0.41 g, 96%; **^1^H NMR** (500 MHz, DMSO-*d*_6_) δ 12.22 (s, 2H, 2NH), 8.41 (s, 3H), 8.17–8.04 (m, 6H), 7.50 (d, *J* = 5.5 Hz, 2H), 7.25–7.22 (m, 2H), 7.13–7.11 (m, 2H), 3.83 (s, 3H, OCH_3_); **^13^C NMR** (125 MHz, DMSO-*d*_6_) δ 182.0, 163.2, 152.5, 137.1, 135.9, 133.3, 126.7, 125.4, 124.0, 122.8, 121.9, 118.9, 115.2 (CN), 114.2, 112.9, 108.4, 56.2 (OCH_3_); **Anal**. Calcd for C_29_H_20_N_4_O (440.51): C, 79.07; H, 4.58; N, 12.72; found: C, 66.98; H, 4.47; N, 12.66.

*4-(3,5-Dimethoxyphenyl)-2,6-bis(1H-indol-3-yl)pyridine-3-carbonitrile (**4f**).* Recrystallized from methanol as yellow crystals; **MP.** 202–204° C; **yield**: 0.4g, 85%; **^1^H NMR** (500 MHz, DMSO-*d*_6_) δ 12.29 (s, 2H, 2NH), 8.41 (s, 2H), 8.15–8.14 (m, 3H), 7.53–7.52 (m, 2H), 7.23 (m, 5H), 6.72 (s, 2H), 3.78, 3.77 (2s, 6H, 2OCH_3_); **^13^C NMR** (125 MHz, DMSO-*d*_6_) δ 181.8, 161.1, 152.5, 137.2, 136.6, 134.5, 126.6, 126.6, 124.1, 123.0, 121.8, 118.1(CN), 114.0, 113.0, 112.4, 108.6, 104.7, 56.0 (2OCH_3_); **Anal**. Calcd for C_30_H_22_N_4_O_2_ (470.53): C, 76.58; H, 4.71; N, 11.91; found: C, 76.51; H, 4.69; N, 11.87.

*4-(3,4,5-Trimethoxyphenyl)-2,6-bis(1H-indol-3-yl)pyridine-3-carbonitrile (**4g**).* Recrystallized from methanol as yellow crystals; **MP.** 133–135 °C; **yield**: 0.4 g, 82%; **^1^H NMR** (500 MHz, DMSO-*d*_6_) δ 12.26 (s, 2H, 2NH), 8.40 (s, 2H), 8.16 (m, 4H), 7.53–7.25 (m, 7H), 3.78, 3.77 (s, 9H, 3OCH_3_); **^13^C NMR** (125 MHz, DMSO-*d*_6_) δ 181.8, 153.4, 152.7, 137.2, 136.2, 128.1, 126.6, 124.1, 122.9, 121.9, 118.6 (CN), 114.1, 113.0, 112.5, 110.5, 108.8, 60.8, and 56.0 (3OCH_3_); **Anal**. Calcd for C_31_H_24_N_4_O_3_ (500.56): C, 74.39; H, 4.83; N, 11.19; found: C, 74.22; H, 4.77; N, 11.01.

*4-(4-Nitrophenyl)-2,6-bis(1H-indol-3-yl)pyridine-3-carbonitrile (**4h**).* Recrystallized from methanol as yellow crystals; **MP.** 249–251°C; **yield**: 0.27 g, 62%; **^1^H NMR** (500 MHz, DMSO-*d*_6_) δ 12.35 (s, 2H, 2NH), 8.49 (s, 1H), 8.39–8.37 (m, 4H), 8.32 (d, *J* = 10 Hz, 1H), 8.20–8.17 (m, 4H), 7.52–7.50 (d, *J* = 10 Hz, 2H), 7.27–7.25 (m, 3H); **^13^C NMR** (125 MHz, DMSO-*d*_6_) δ 192.8, 181.3, 149.8, 149.2, 139.1, 137.3, 131.6, 131.1, 126.5, 124.7, 124.5, 124.2, 123.6, 121.8, 117.3 (CN), 115.6, 113.9, 113.1; **Anal**. Calcd for C_28_H_17_N_5_O_2_ (455.48): C, 73.84; H, 3.76; N, 15.38; found: C, 73.79, H, 3.69; N, 15.22.

*4-(4-(Dimethylamino)phenyl)-2,6-bis(1H-indol-3-yl)pyridine-3-carbonitrile (**4i**).* Recrystallized from methanol as yellow crystals; **MP.** 271–273 °C; **yield**: 0.27 g, 60%; **^1^H NMR** (500 MHz, DMSO-*d*_6_) δ 12.07 (s, 2H, 2NH), 8.38 (s, 2H), 8.14–8.09 (m, 3H), 7.96 (d, *J* = 15 Hz, 2H), 7.50 (m, 2H), 7.30–7.10 (m, 3H), 6.81 (m, 3H), 3.05 (2s, 6H, 2CH_3_); **^13^C NMR** (125 MHz, DMSO-*d*_6_) δ 182.1, 153.7, 153.3, 136.9, 134.6, 133.8, 126.9, 123.7, 122.5, 122.0, 120.5, 119.7, 114.5 (CN), 112.8, 112.2, 102.6, 40.0 (2CH_3_); **Anal**. Calcd for C_30_H_23_N_5_ (453.55): C, 79.45; H, 5.11; N, 15.44; found: C, 79.44; H, 5.22; N, 15.32.

*4-(4-Hydroxyphenyl)-2,6-bis(1H-indol-3-yl)pyridine-3-carbonitrile (**4j**).* Recrystallized from methanol as yellow crystals; **MP.** 150–152 °C; **yield**: 0.28 g, 67%; **^1^H NMR** (500 MHz, DMSO-*d*_6_) δ 12.15 (s, 2H, 2NH), 8.38 (s, 2H), 8.14–8.11 (m, 4H), 7.97–7.95 (m, 4H), 7.51–7.50 (m, 2H), 7.62–7.21 (m, 2H), 6.93–6.91 (m, 2H); **^13^C NMR** (125 MHz, DMSO-*d*_6_) δ 182.1, 173.9, 162.5, 152.9, 137.1, 135.6, 134.1, 133.8, 126.7, 123.9, 122.7, 121.9, 119.2, 116.9, 116.7 (CN), 114.2, 112.9, 107.0, 93.5; **Anal**. Calcd for C_28_H_18_N_4_O (426.48): C, 78.86; H, 4.25; N, 13.14; found: C, 78.80; H, 4.11; N, 13.01.

*General procedure for the preparation of 2,6-bis(1H-indol-3-yl)-4-(benzo-furan-2-yl)pyridin-5-carbonitriles **7a**–**e**.*

**Method A**. A mixture of 3-(1*H*-indol-3-yl)-3-oxopropanenitrile (**1**, 1.0 mmol) and the appropriate *N*-substituted indole-3-aldehydes (**5**, 1.0 mmol) in glacial acetic acid (20 mL) was stirred at 120 °C for 1 h. Then, the 2-acetyl benzofuran **6** (1.0 mmol) and ammonium acetate (10.0 mmol) were added to the reaction mixture and stirred for 2 h at 120 °C. At the end of the reaction (as monitored by TLC), the reaction mixture was cooled to room temperature and quenching with ice water. The produced solid was filtered off, air-dried, and subsequently recrystallized from the proper solvent to give the pure products **7**.

**Method B**. **Cyclization of compounds 8a–e**. A mixture of chalcones (**8**, 1.0 mmol), 3-(1*H*-indol-3-yl)-3-oxopropanenitrile (**1**, 1.0 mmol), and ammonium acetate (10.0 mmol) in glacial acetic acid (20 mL) was stirred at 120 °C for 7–9 h. After the completion of the reaction (as monitored by TLC), the reaction mixture was cooled to room temperature and quenching with ice water. The formed solid was filtered off, air-dried, and recrystallized from the proper solvent to give the pure products **7**.

*6-(Benzofuran-2-yl)-2,4-bis(1H-indol-3-yl)pyridine-3-carbonitrile (**7a**).* Recrystallized from ethanol as yellow crystals; **MP.** 259–261 °C; **yield**: method A, 0.22 g, 50%; method B, 0.23 g, 50%; **^1^H NMR** (500 MHz, DMSO-*d*_6_) δ 11.87 (s, 2H, 2NH), 8.56–8.54 (m, 3H), 8.39–8.37 (m, 3H), 7.83–7.81 (m, 1H), 7.70 (d, *J* = 10 Hz, 1H), 7.57 (d, *J* = 10 Hz, 1H), 7.43 (m, 3H), 7.33–7.25 (m, 4H); **^13^C NMR** (125 MHz, DMSO-*d*_6_) δ 156.8, 155.6, 154.3, 150.8, 144.2, 136.9, 128.9, 128.7, 127.0, 126.3, 124.3, 123.1, 123.0, 122.4, 121.6, 115.2 (CN), 112.9, 112.6, 112.2, 108.3, 102.8; **Anal**. Calcd for C_30_H_18_N_4_O (450.50): C, 79.98; H, 4.03; N, 12.44; found: C, 80.01; H, 3.99; N, 12.43. 

*6-(Benzofuran-2-yl)-4-(1-ethyl-1H-indol-3-yl)-2-(1H-indol-3-yl)pyridine-3-carbonitrile (**7b**).* Recrystallized from ethanol as yellow crystals; **MP.** 194–196 °C; **yield**: method A, 0.35 g, 75%; method B, 0.36 g, 75%; **^1^H NMR** (500 MHz, DMSO-*d*_6_) δ 12.12 (s, 1H, NH), 8.59–8.58 (m, 3H), 8.47 (s, 1H), 8.23–8.21 (d, *J* = 10 Hz, 1H), 7.98 (d, *J* = 10 Hz, 1H), 7.62–7.54 (m, 3H), 7.30–7.24 (m, 7H), 4.36 (q, 2H), 1.4 (t, 3H); **^13^C NMR** (125 MHz, DMSO-*d*_6_) δ 181.2, 145.0, 136.9, 134.3, 133.4, 124.0, 123.8, 122.7, 122.5, 122.1, 119.4, 112.9, 111.7, 102.1, 40.1, 15.6; **Anal**. Calcd for C_32_H_22_N_4_O (478.56): C, 80.32; H, 4.63; N, 11.71; found: C, 80.30; H, 4.55; N, 11.80.

*6-(Benzofuran-2-yl)-4-(1-benzyl-1H-indol-3-yl)-2-(1H-indol-3-yl)pyridine-3-carbonitrile (**7c**).* Recrystallized from acetone as yellow crystals; **MP.** 181–183 °C; **yield**: method A, 0.31 g, 60%; method B, 0.32 g, 60%; **^1^H NMR** (500 MHz, DMSO-*d*_6_) δ 11.87 (s, 1H, NH), 8.65–8.51 (m, 2H), 8.39–8.38 (m, 2H), 8.31 (m, 1H), 8.20–8.16 (m, 1H), 8.09–8.01 (m, 1H), 7.89–7.77 (m, 3H), 7.76–7.63 (m, 2H), 7.63–6.72 (m, 9H), 5.49 (s, 1H), 5.38 (s, 1H); **^13^C NMR** (125 MHz, DMSO-*d*_6_) δ 181.0, 155.6, 154.3, 150.8, 144.2, 137.0, 136.9, 129.2, 129.0, 128.9, 127.7, 127.5, 127.0, 124.3, 123.1, 121.6, 115.2 (CN), 112.6, 108.3, 102.8, 49.9, 49.6; **Anal**. Calcd for C_37_H_24_N_4_O (540.63): C, 82.20; H, 4.47; N, 10.36; found: C, 82.01; H, 4.50; N, 10.22.

*6-(Benzofuran-2-yl)-4-(1-benzoyl-1H-indol-3-yl)-2-(1H-indol-3-yl)pyridine-3-carbonitrile (**7d**).* Recrystallized from ethanol as yellow crystals; **MP.** 116–118 °C; **yield**: method A, 0.3 g, 55%; method B, 0.3 g, 54%; **^1^H NMR** (500 MHz, DMSO-*d*_6_) δ 11.84 (s, 1H, NH), 8.57–8.55 (d, *J* = 10 Hz, 1H), 8.50–8.41 (m, 1H), 8.28–8.18 (m, 1H), 8.10–8.00 (m, 1H), 7.78–7.24 (m, 17H); **Anal**. Calcd for C_37_H_22_N_4_O_2_ (554.61): C, 80.13; H, 4.00; N, 10.10; found: C, 80.01; H, 3.98; N, 9.98.

*6-(Benzofuran-2-yl)-2-(1H-indol-3-yl)-4-(1-(phenylsulfonyl)-1H-indol-3-yl)pyridine-3-carbonitrile (**7e**).* Recrystallized from ethanol as yellow crystals; **MP.** 247–249 °C; **yield**: method A, 0.5 g, 85%; method B, 0.47 g, 80%; **^1^H NMR** (500 MHz, DMSO-*d*_6_) δ 11.90 (s, 1H), 8.56 (dd, *J* = 11.1, 4.0 Hz, 2H), 8.46 (d, *J* = 2.9 Hz, 1H), 8.09 (t, *J* = 8.5 Hz, 2H), 8.06 (d, *J* = 8.4 Hz, 1H), 7.96 (s, 1H), 7.89 (s, 1H), 7.80 (dt, *J* = 18.3, 9.1 Hz, 2H), 7.74–7.67 (m, 2H), 7.64–7.58 (m, 2H), 7.54 (t, *J* = 7.8 Hz, 2H), 7.43 (tdd, *J* = 25.4, 16.6, 9.1 Hz, 3H), 7.29 (ddt, *J* = 12.8, 7.1, 6.7 Hz, 2H); **^13^C NMR** (125 MHz, DMSO-*d*_6_) δ 158.45, 155.68, 154.25, 150.55, 146.94, 135.61, 130.56, 129.63, 128.71, 128.57, 128.28, 127.72, 127.60, 127.06, 126.62, 126.31, 126.27, 124.93, 124.33, 123.10, 123.06, 123.02, 122.39, 121.65, 121.10, 119.38, 118.66, 115.82 (CN), 114.02, 113.12, 112.67, 112.27, 108.78, 102.58; **Anal**. Calcd for C_36_H_22_N_4_O_3_S (590.66): C, 73.21; H, 3.75; N, 9.49; S, 5.43; found: C, 73.11; H, 3.66; N, 9.50; S, 5.51.

*General Procedure for the preparation of (E)-1-(benzofuran-2-yl)-3-(N-substituted-indol-3-yl)prop-2-en-1-one*: To a solution of 2-acetyl benzofuran (**6**, 1.0 mmol) in absolute ethanol (20 mL) containing few drops of piperidine (0.5 mL), N-substituted indole-3-aldehydes (**5**, 1.0 mmol) was added. The reaction mixture was heated under reflux for 1 h. The precipitate formed from the heat was filtered off and dried as pure product **8** without further crystallization. 

*(E)-1-(Benzofuran-2-yl)-3-(1H-indol-3-yl)prop-2-en-1-one (**8a**)* Yellow powder; yield 60%; MP 184–186 °C (reported m.p. 184 °C) [30]. 

*(E)-1-(Benzofuran-2-yl)-3-(1-ethyl-1H-indol-3-yl)prop-2-en-1-one (**8b**).* Yellow crystals; **MP.** 149–151 °C; **yield**: 0.23 g, 74%; **^1^H NMR** (500 MHz, DMSO-*d*_6_) δ 8.19 (s, 1H), 8.14 (d, *J* = 10 Hz, 1H), 8.07 (d, *J* = 5 Hz, 1H), 8.04 (d, *J* = 5 Hz, 1H), 7.82 (d, *J* = 10 Hz, 1H), 7.72 (d, *J* = 10 Hz, 1H), 7.59–7.51 (m, 3H), 7.35–7.28 (m, 3H), 4.25 (q, 2H), 1.39 (t, 3H); **Anal**. Calcd for C_21_H_17_NO_2_ (315.37): C, 79.98; H, 5.43; N, 4.44; found: C, 80.01; H, 5.51; N, 4.32.

*(E)-1-(Benzofuran-2-yl)-3-(1-benzyl-1H-indol-3-yl)prop-2-en-1-one (**8c**).* Recrystallized from ethanol as yellow crystals; **MP.** 206–208 °C; **yield**: 0.2 g, 58%; **^1^H NMR** (500 MHz, DMSO-*d*_6_) δ 8.31 (s, 1H), 8.16–7.26 (m, 16H), 5.48 (s, 2H, CH_2_); **Anal**. Calcd for C_26_H_19_NO_2_ (377.44): C, 82.74; H, 5.07; N, 3.71; found: C, 82.66; H, 4.99; N, 3.68.

*(E)-1-(Benzofuran-2-yl)-3-(1-benzoyl-1H-indol-3-yl)prop-2-en-1-one (**8d**).* Recrystallized from ethanol as yellow crystals; **MP.** 217–219 °C; **yield**: 0.23 g, 58%; **^1^H NMR** (500 MHz, DMSO-*d*_6_) δ 8.23–8.05 (m, 5H), 7.85 (d, *J* = 5.9 Hz, 1H), 7.75 (d, *J* = 5.9 Hz, 1H), 7.55 (dd, *J* = 40.2, 13.0 Hz, 8H), 7.37 (s, 1H), 7.25 (s, 1H); **Anal**. Calcd for C_26_H_17_NO_3_ (391.43): C, 79.78; H, 4.38; N, 3.58; found: C, 79.82; H, 4.44; N, 3.60. 

*(E)-1-(Benzofuran-2-yl)-3-(1-(phenylsulfonyl)-1H-indol-3-yl)prop-2-en-1-one (**8e**)*. Recrystallized from ethanol as yellow crystals; **MP.** 243–245 °C; **yield**: 0.36 g, 85%; **^1^H NMR** (500 MHz, DMSO-*d*_6_) δ 8.21–8.06 (m, 3H), 8.05–8.02 (m, 2H), 7.96 (ddd, *J* = 23.8, 14.1, 6.2 Hz, 2H), 7.83 (d, *J* = 7.9 Hz, 1H), 7.73 (d, *J* = 8.3 Hz, 1H), 7.66–7.34 (m, 5H), 7.27–7.21 (m, 3H).; **Anal**. Calcd for C_25_H_17_NO_4_S (427.47): C, 70.24; H, 4.01; N, 3.28; S, 7.50; found: C, 70.22; H, 3.98; N, 3.33; S, 7.60.

### 3.2. Biological Studies

#### Antimicrobial Activity

The antimicrobial activity of target compounds was evaluated to be in vitro against a variety of pathogenic microorganisms, namely *Staphylococcus aureus* ATCC 6538 (G+ve), *Escherichia coli* ATCC 25922 (G-ve), *Candida albicans* ATCC 10231 (yeast), and *Aspergillus niger* NRRL A-326 (fungus). The test samples were prepared by dissolving 5 mg of the test compounds in 2 mL of DMSO, and then 100 μL (containing 250 μg) was used to evaluate their antimicrobial activities by cup plate diffusion method using the test microbes [39]. Nutrient agar plates were heavily inoculated uniformly with 100 μL of 10^5^–10^6^ cells/mL in case of bacteria and yeast. Potato Dextrose agar plate seeded by 100 μL of the fungal inoculum was used to evaluate the antifungal activity. A 1 cm diameter hole was made in media by gel cutter (Cork borer) in a sterile condition. One drop of melted agar was poured into the bottom of the hole and allowed to solidify to make a base layer. A total of 100 μL of dissolved samples were poured into the holes. The plates were kept at a low temperature (4 °C) for 2–4 h, to allow maximum diffusion. The plates were incubated at 37 °C for 24 h for bacteria, and at 30 °C for 48 h in an upright position to prevent the scattering of liquid samples and allow maximum growth of the organisms. The antimicrobial activity of the tested samples was investigated by measuring the diameter of the inhibition zones expressed in millimeters (mm) [40]. The experiment was carried out twice and the mean of the reading was recorded. Neomycin and cycloheximide were used as a positive control for bacterial and fungal test microbes at the same concentrations.

### 3.3. MICs and MBCs Evaluation 

MIC was performed using *S. aureus* ATCC 6538, Gram-positive bacteria, and *E. coli* ATCC 25922, Gram-negative bacteria, as tested microbes that are grown on a Mueller Hinton medium. Test microbes were cultivated in 100 mL bottles with each test at 35 °C for 24 h. Cells were obtained by centrifugation (4000 rpm) under a sterile condition at 4 °C for 15 min. The cells were washed using sterile saline until the supernatant was clear. Cells with an optical density of 0.5 to 1 (at 550 nm) giving an actual number of colony-forming units of 5 × 10^6^ cfu/mL were obtained. Resazurin solution was prepared by dissolving 270 mg tablet in 40 mL of sterile distilled water. Then, 96-well sterile microplates were prepared. Then, 50 μL of test material in DMSO was pipetted into the first row of the plate. To all other wells, 50 μL of broth medium was added. Two-fold serial dilutions were performed. Then, 10 μL of resazurin indicator solution was added, 10 μL of bacterial suspension was added to each well. The plates were prepared in duplicate and placed in an incubator set at 37 °C for 18–24 h. Any colour changes from purple to pink or colourless were recorded as positive. The lowest concentration at which colour change occurred was taken as the MIC value. MBC has been performed by streaking of the two concentrations higher than MIC and the plates exhibiting no growth were considered as MBC [41]. 

### 3.4. Inhibition of Biofilm Formation (Crystal Violet Method)

Bacterial strains were incubated in test tubes with TSB (5 mL) containing 2% *w*/*v* glucose at 37 °C for 24 h. After that, the bacterial suspensions were diluted to achieve turbidity equivalent to a 0.5 McFarland standard. The diluted suspension (2.5 μL) was added to each well of a single cell culture polystyrene sterile, flat-bottom 96-well plate filled with TSB (200 μL) with 2% *w*/*v* glucose. Sub-MIC concentration values of compounds **4b**, **7a**, **7c**, **7d**, and **8e** were directly added to the wells to reach concentrations ranging from 100 to 0.1 μM to assess BIC_50_ values that are, the concentration at which the percentage of inhibition of biofilm formation is equal to 50%. Plates were incubated at 37 °C for 24 h. After biofilm growth, the content of each well was removed, wells were washed twice with sterile NaCl 0.9% and stained with 200 μL of 0.1% *w*/*v* crystal violet solution for 15 min at 37 °C. The excess solution was removed, and the plate was washed twice, using tap water. A volume of 200 μL of ethanol was added to each stained well to solubilize the dye [13]. Optical density (O.D.) was read at 600 nm using a microplate reader (GloMax^®^-Multi Detection System, Milan, Italy). The experiments were run at least in triplicates, and three independent experiments were performed. The percentage of inhibition was calculated through the formula:

% Inhibition = (OD growth − OD sample/OD growth control) × 100(1)

### 3.5. Release of Cellular Sugars and Proteins 

To detect the leakage of reducing sugars and proteins through bacterial membranes, the pure compounds were added into 10 mL of cultures with a final concentration of 250 μg/mL, and 20 μL bacterial cell suspension giving a final concentration of 10^6^ cfu/mL of both *Staphylococcus aureus* and *Escherichia coli.* Control experiments were conducted without the addition of compounds. The cultures were incubated at 37 °C with shaking at 150 rpm. The sample was centrifuged at 12,000 rpm, the supernatant liquid was frozen at −30 °C immediately and then the concentrations of reducing sugars and proteins were determined as soon as possible [42,43].

### 3.6. Molecular Docking

#### 3.6.1. Target Preparation

The crystallographic structures of Thymidylate kinase (TMPK), DNA gyrase subunit B, and DNA topoisomerase IV subunit B were retrieved from the protein data bank at http://www.rscb.org./pdb (accessed on 6 June 2021) using 4QGG, 6F86, 4HZ5 codes, respectively, and were selected as a target in the modeling study. Water molecules were removed, hydrogen atoms and partial charges were added, then Gaussian Contact surface around the binding sites were drawn using MOE 2008.10 (Molecular Operating Environment, http://www.chemcomp.com (accessed on 5 May 2013).

#### 3.6.2. Ligand Preparation

The most active antimicrobial compounds **4b**, **7a**, **7c**, **7d**, and **8e** were generated using ChemDraw Ultra 12.0, converted into 3D and prepared by the MOE program. The structures were minimized using the MMFF94x force field (eps= r, Cutoff until the RMSD gradient of 0.01 kcal mol^−1^ Å^−1^ was reached). The protonation state of each compound was assigned based on the neutral pH. All the compounds were imported into the MOE database to be used in docking. 

#### 3.6.3. Docking Procedures

Docking of the studied structures was carried out using MOE 2008.10 program. First, the validation of the docking protocol was carried out by running simulation studies for the co-crystallized ligands of all target proteins. All the re-docked ligands showed low RMSD less than 2 Å−1 indicated the validity of the applied protocol. Molecular docking and free binding energies were calculated. The placement was selected to be Triangle Matcher docking, London dG for rescoring function 1, and refinement with Force field. The simulation process created 30 poses, which were sorted according to the lowest energy. The five selected compounds were docked into the binding site of each enzyme and free binding energies were calculated.

## 4. Conclusions

In conclusion, a new series of bis(indolyl)pyridines as analogues of the marine alkaloid nortopsentin was designed using an efficient and simple pathway of a one-pot four-component condensation reaction. The synthesized compounds were assessed for their in vitro antimicrobial (MIC, MBC), anti-biofilm properties. Moreover, reducing sugars and proteins were measured as a tool to explain the effect of active compounds on test microbes. Our obtained data revealed that the selected compounds (**4b**, **7a**, **7c**, **7d**, and **8e**) exhibited antibiofilm formation against Gram-positive and Gram-negative bacteria. Therefore, these compounds could be used to tolerate drug-resistant bacteria. The disturbance effect of these compounds was deduced from their effect on intact bacterial cells leading to the release of biomolecules such as reducing sugars and proteins. The mechanisms of action of the most active compounds **4b**, **7a**, **7c**, **7d** and **8e** were investigated against different bacterial enzymes using the MOE program in comparison to their native ligands. The results obtained revealed two compounds **7c** and **7d** could be employed for the future preparation of pharmaceutical formula containing bis-indole to overcome evolved multidrug resistance behaviors by microbes.

## Figures and Tables

**Table 1:** In vitro antimicrobial activity of the new synthesized compounds against a variety of pathogenic microorganisms ^a^.

Compd. No ^b^	Inhibition Zone (ф mm)			
	*S. aureus* ATCC 6538	*E. coli* ATCC 25922	*C. albicans* ATCC 10231	*A. niger* NRRL A-326
**4a**	0	0	13	29
**4b**	20	15	16	26
**4c**	15	13	13	20
**4d**	16	0	15	18
**4e**	14	16	14	16
**4f**	14	0	12	19
**4g**	15	0	12	18
**4h**	16	14	0	16
**4i**	14	0	12	17
**4j**	0	0	0	0
**7a**	18	0	17	13
**7b**	0	12	13	14
**7c**	17	13	12	0
**7d**	36	12	27	14
**7e**	12	14	0	13
**8a**	12	13	14	12
**8b**	0	0	14	0
**8c**	0	0	0	0
**8d**	0	0	13	14
**8e**	21	14	22	14
neomycin	27	25	28	0
cyclohexamide	0	0	0	22

a, cup diffusion method; b, concentration at 250 μg/100 μL.

**Table 2:** The minimum inhibitory concentrations (MICs), and minimum bactericidal concentrations (MBCs) of the most active selected compounds.

Compounds	Pathogenic Microorganisms					
	*S. aureus* ATCC 6538		*E. coli* ATCC 25922		*C. albicans* ATCC 10231	
	MIC (μg/mL)	MBC (μg/mL)	MIC (μg/mL)	MBC (μg/mL)	MIC (μg/mL)	MBC (μg/mL)
**4b**	78.125	312.5	312.5	1250	78.13	312.5
**7a**	39.063	625	312.5	625	39.06	156.25
**7c**	312.5	625	625	1250	39.06	78.13
**7d**	9.766	19.53	312.5	1250	19.53	19.53
**8e**	19.53	19.53	312.5	625	19.53	39.06

**Table 3:** Biofilm inhibition (%) and (IC_50_) from *Staphylococcus aureus* and *Escherichia coli cultures* treated with the most active selected compounds.

Compds	Biofilm Inhibition (IC_50_)		Biofilm Inhibition (%)	
	*Staphylococcus aureus*	*Escherichia coli*	*Staphylococcus aureus*	*Escherichia coli*
**4b**	304.42 ± 0.32 ^b^	56.52 ± 0.76 ^d^	76.59 ± 0.34 ^c^	91.02 ± 0.65 ^a^
**7a**	103.52 ± 0.76 ^e^	431.21 ± 0.68 ^b^	76.21 ± 0.45 ^c^	65.33 ± 0.61 ^c^
**7c**	449.81 ± 0.66 ^a^	729.32 ± 0.76 ^a^	67.52 ± 0.65 ^d^	37.51 ± 0.69 ^d^
**7d**	199.32 ± 0.71 ^c^	266.91 ± 0.17 ^c^	82.06 ± 0.66 ^a^	67.61 ± 0.57 ^b^
**8e**	169.63 ± 0.81 ^d^	54.65 ± 0.62 ^e^	81.08 ± 0.84 ^b^	90.88 ± 0.72 ^a^

Values are expressed as means ± SE; *n* = 3 for each group. Mean values within a column not sharing common superscript letters (a, b, c, d, e) are significantly different, (*p* ≤ 0.05).

**Table 4:** Reducing sugars and proteins released from *Staphylococcus aureus* and *Escherichia coli cultures* treated with the most active selected compounds compared to untreated cultures (control).

Compds	Reducing Sugars (μg/mL)		Proteins Release (μg/mL)	
	*Staphylococcus aureus*	*Escherichia coli*	*Staphylococcus aureus*	*Escherichia coli*
**Control**	172.365 ± 2.81 ^f^	116.198 ± 1.69 ^f^	158.9 ± 2.09 ^f^	194.5 ± 1.68 ^f^
**4b**	249.533 ± 2.45 ^d^	208.533 ± 1.23 ^d^	389.95 ± 5.71 ^e^	405.9 ± 7.71 ^e^
**7a**	327.2 ± 3.88 ^a^	227.318 ± 3.11 ^c^	480.8 ± 5.25 ^c^	562.9 ± 4.59 ^a^
**7c**	209.818 ± 3.19 ^e^	234.368 ± 2.33 ^a^	607.4 ± 2.46 ^a^	437.8 ± 1.46 ^d^
**7d**	306.203 ± 5.36 ^b^	190.368 ± 2.47 ^e^	462.5 ± 16.14 ^d^	453.4 ± 1.54 ^c^
**8e**	254.533 ± 2.54 ^c^	230.7 ± 2.37 ^b^	540.9 ± 5.76 ^b^	523.8 ± 3.64 ^b^

Values are expressed as means ± SE; *n* = 3 for each group. Mean values within a column not sharing common superscript letters (a, b, c, d, e, f) are significantly different, (*p* ≤ 0.05).

**Table 5:** Docking results of the most antimicrobial active compounds **4b**, **7a**, **7c**, **7d**, **8e**, the native thymidylate kinase (ID: 4QGG), the DNA gyrase B (ID: 6F86), and the DNA topoisomerase IV subunit B (ID: 4HZ5).

Comp. No.	Moieties from the Compound	Amino Acid Residues	Type of Interaction Distance	Score Kcal/mol
**Thymidylate kinase (ID: 4QGG)**				
**Native ligand**	NH	Gln101	H-don (1.68, 2.85)	−24.34 RMSD: 1.42
	CO	Arg48, Arg70, Ser97	H-acc (2.24, 2.79, 2.75)	
**4b**	Indole moiety	Lys15, Arg92	π-Cation	−22.43
	Indole moiety and Pyridine ring	Phe66, Tyr100	π-π	
**7a**	Indole moiety and benzofuran	Arg48	π-Cation	−15.52
	Indole moiety	Arg36	π-Cation	
**7c**	Indole moiety and benzofuran	Arg92	π-Cation	−23.01
	Benzofuran	Lys15	π-Cation	
**7d**	CN and CO	Arg92	H-acc (2.88, 2.51)	−25.36
	Indole moiety	Arg92	π-Cation	
**8e**	CO	Arg48	H-acc (2.51)	−20.61
	benzofuran	Arg36	π-Cation	
**BNA gyrase B (ID: 6F86)**				
**Native ligand**	NH	Asp73	H-don (1.85)	−22.84 RMSD: 1.65
	NH	Gly77	H-don (2.10)	
	Pyridine ring	Arg76	π-Cation	
**4b**	Pyridine ring	Arg76	π-Cation	−14.64
**7a**	-	-	-	−19.35
**7c**	CN	Arg76	H-acc (2.93)	−22.31
**7d**	Indole moiety	Arg76	π-Cation	−16.90
**8e**	Indole moiety	Arg76	π-Cation	−7.77
**DNA topoisomerase IV subunit B (ID: 4HZ5)**				
**Native ligand**	Pyridine	Arg79	π-Cation	−14.90 RMSD: 0.84
**4b**	Pyridine	Arg79	π-Cation	−20.85
	Indole moiety	Arg130	π-Cation	
**7a**	CN	Arg138	H-acc (3.21)	−19.09
	Indole moiety	Arg79	π-Cation	
	Benzofuran	His118	π-Cation	
**7c**	Indole moiety	Arg79	π-Cation	−24.90
	Benzofuran	His118	π-Cation	
	NH	Ser56	H-don (2.23)	
**7d**	NH	Ser56	H-don (2.08)	−26.53
	Benzofuran	His118	π-Cation	
	Indole moiety	Arg79	π-Cation	
**8e**	CO	GLY 80	H-acc (2.87)	−23.80
	SO_2_	ARG 138	H-acc (2.47, 2.90)	
	Indole moiety	Arg79	π-Cation	

## Data Availability

Not applicable.

## Supplementary Materials

The following are available online, ^1^H and ^13^C NMR spectral data of the prepared compounds.

## Sample Availability

Samples of the compounds are available from the authors.
