# Supplementary material for: Synthesis, Molecular Docking, and Biofilm Formation Inhibitory Activity of Bis(Indolyl)Pyridines Analogues of the Marine Alkaloid Nortopsentin
Source: Molecules. 2021 Jul 6;26(14):4112. doi: 10.3390/molecules26144112 (PMC8304590; doi:10.3390/molecules26144112)
Supplement: Supplementary file 1 [file molecules-26-04112-s001.zip › molecules-1278653-supplementary.pdf]

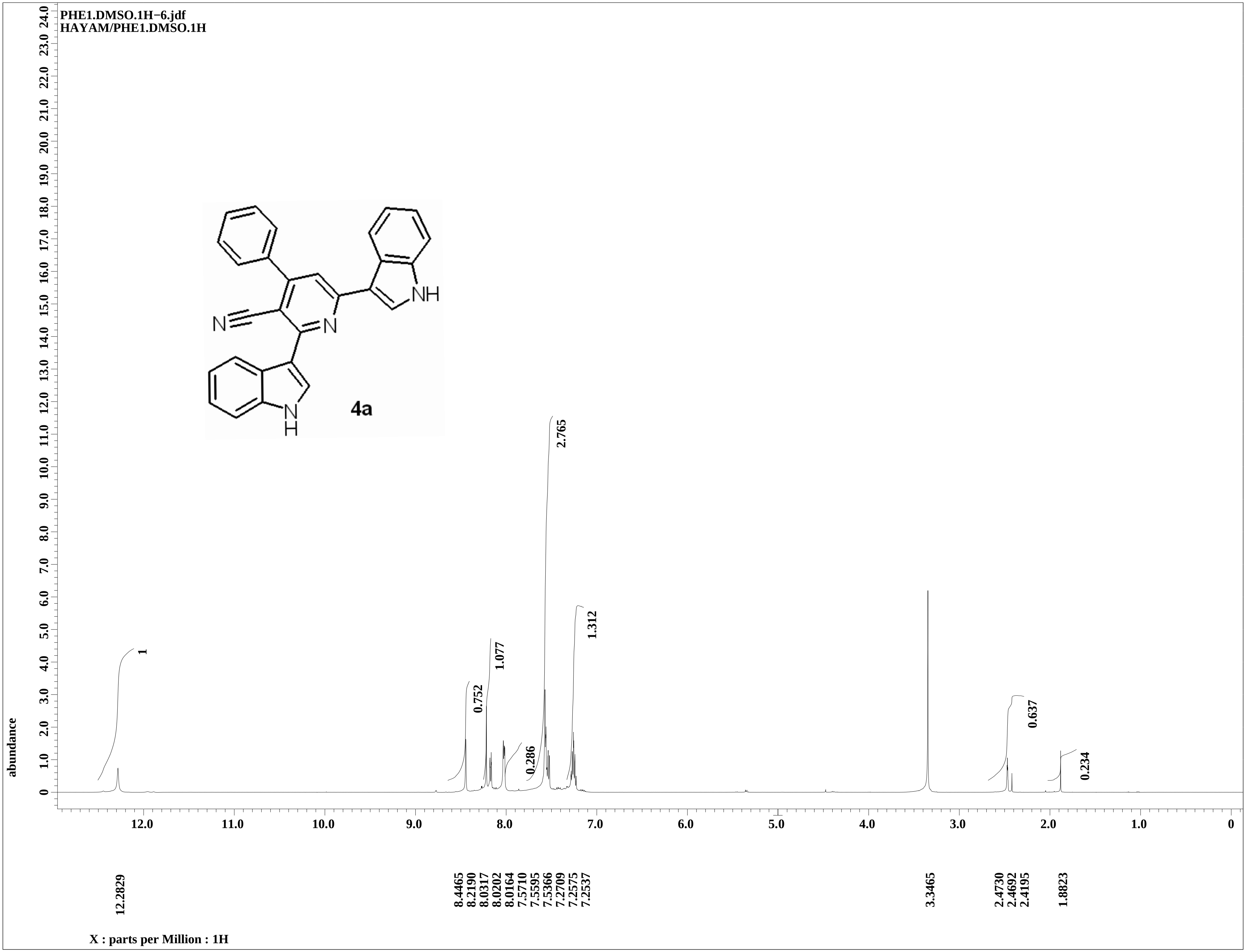
PHE1.DMSO.1H-6.jdf
HAYAM/PHE1.DMSO.1H
4a
abundance
1
0.752
1.077
0.286
2.765
1.312
0.637
0.234
12.2829
8.4465
8.2190
8.0317
8.0202
8.0164
7.5710
7.5595
7.5366
7.2709
7.2575
7.2537
3.3465
2.4730
2.4692
2.4195
1.8823
X : parts per Million : 1H

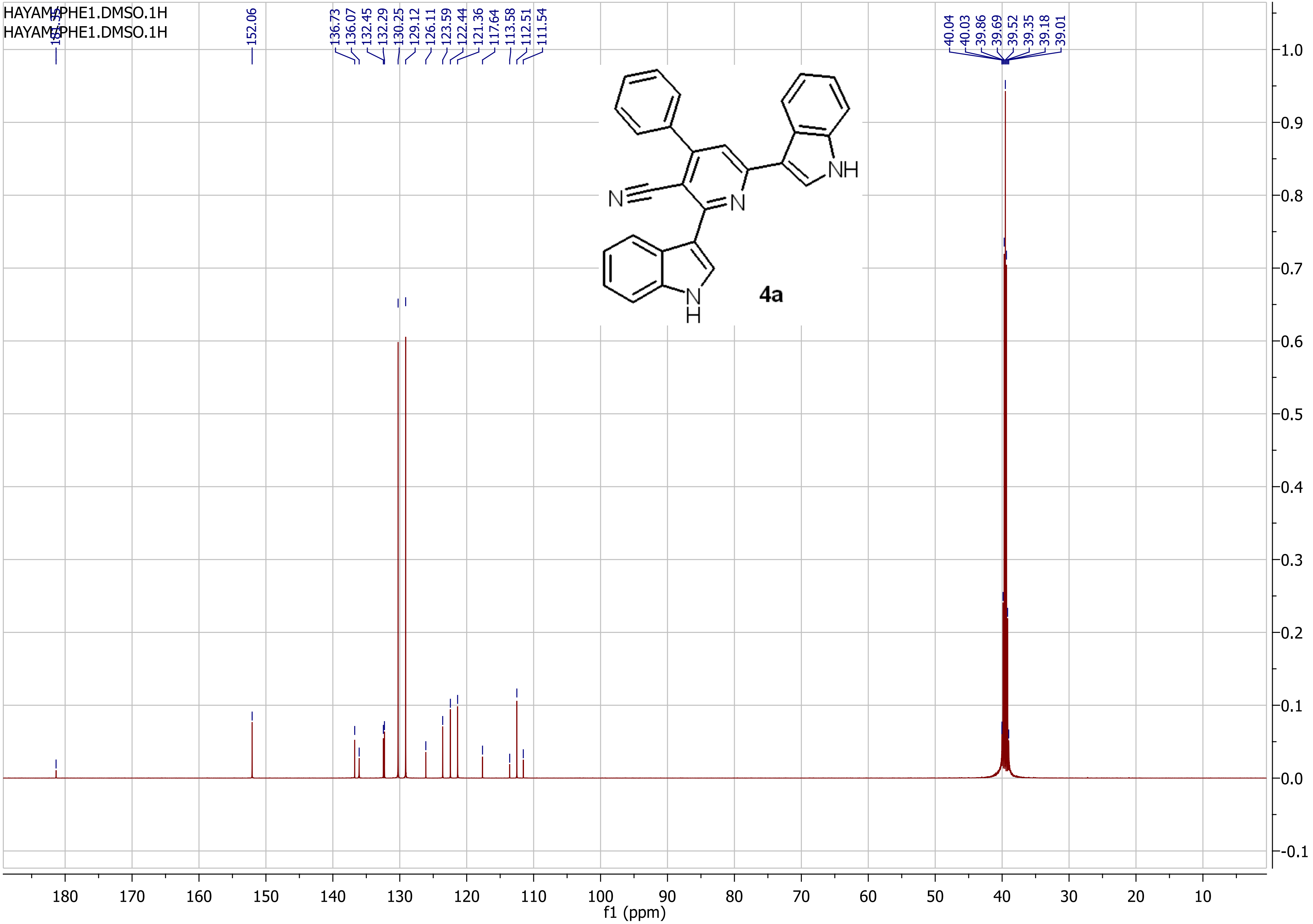
HAYAM/PHE1.DMSO.1H
HAYAM/PHE1.DMSO.1H
152.06
136.73
136.07
132.45
132.29
130.25
129.12
126.11
123.59
122.44
121.36
117.64
113.58
112.51
111.54
40.04
40.03
39.86
39.69
39.52
39.35
39.18
39.01
N
NH
N
H
4a
f1 (ppm)

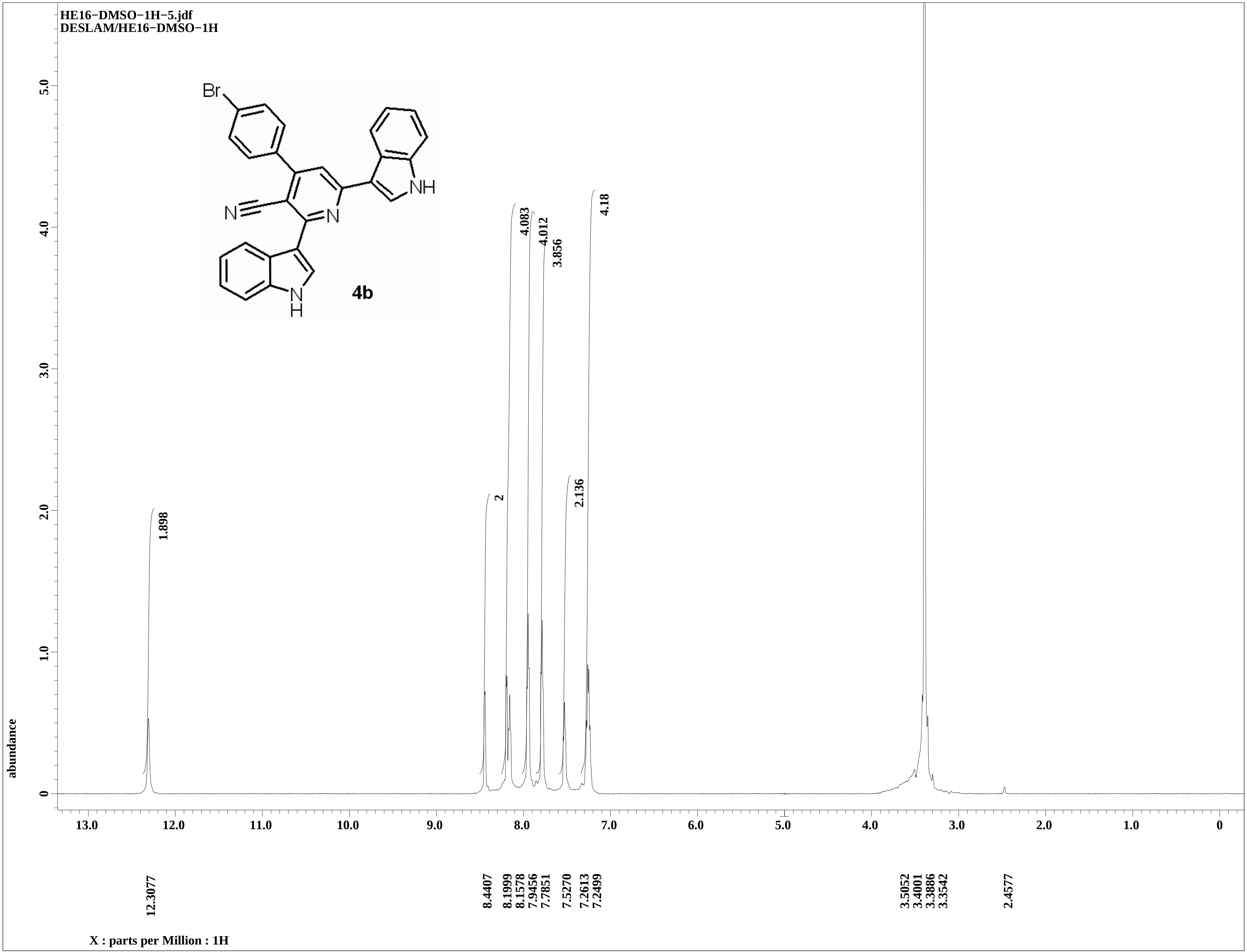
HE16−DMSO−1H−5.jdf
DESLAM/HE16−DMSO−1H
Br
N
NH
N
H
4b
1.898
2
4.083
4.012
3.856
2.136
4.18
abundance
0
1.0
2.0
3.0
4.0
5.0
13.0
12.0
11.0
10.0
9.0
8.0
7.0
6.0
5.0
4.0
3.0
2.0
1.0
0
12.3077
8.4407
8.1999
8.1578
7.9456
7.7851
7.5270
7.2613
7.2499
3.5052
3.4001
3.3886
3.3542
2.4577
X : parts per Million : 1H

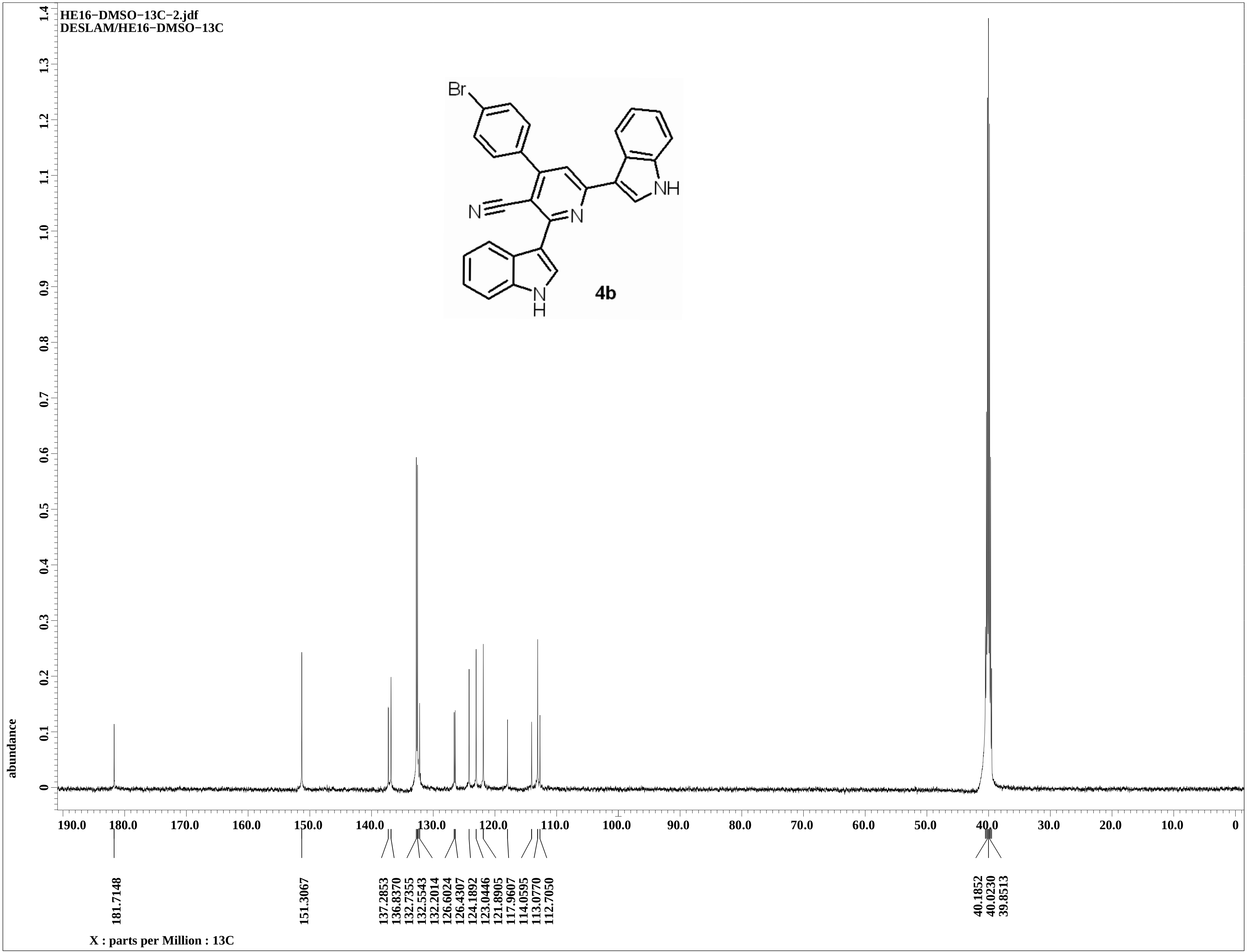
HE16−DMSO−13C−2.jdf
DESLAM/HE16−DMSO−13C
abundance
Br
N
NH
N
H
4b
181.7148
151.3067
137.2853
136.8370
132.7355
132.5543
132.2014
126.6024
126.4307
124.1892
123.0446
121.8905
117.9607
114.0595
113.0770
112.7050
40.1852
40.0230
39.8513
X : parts per Million : 13C

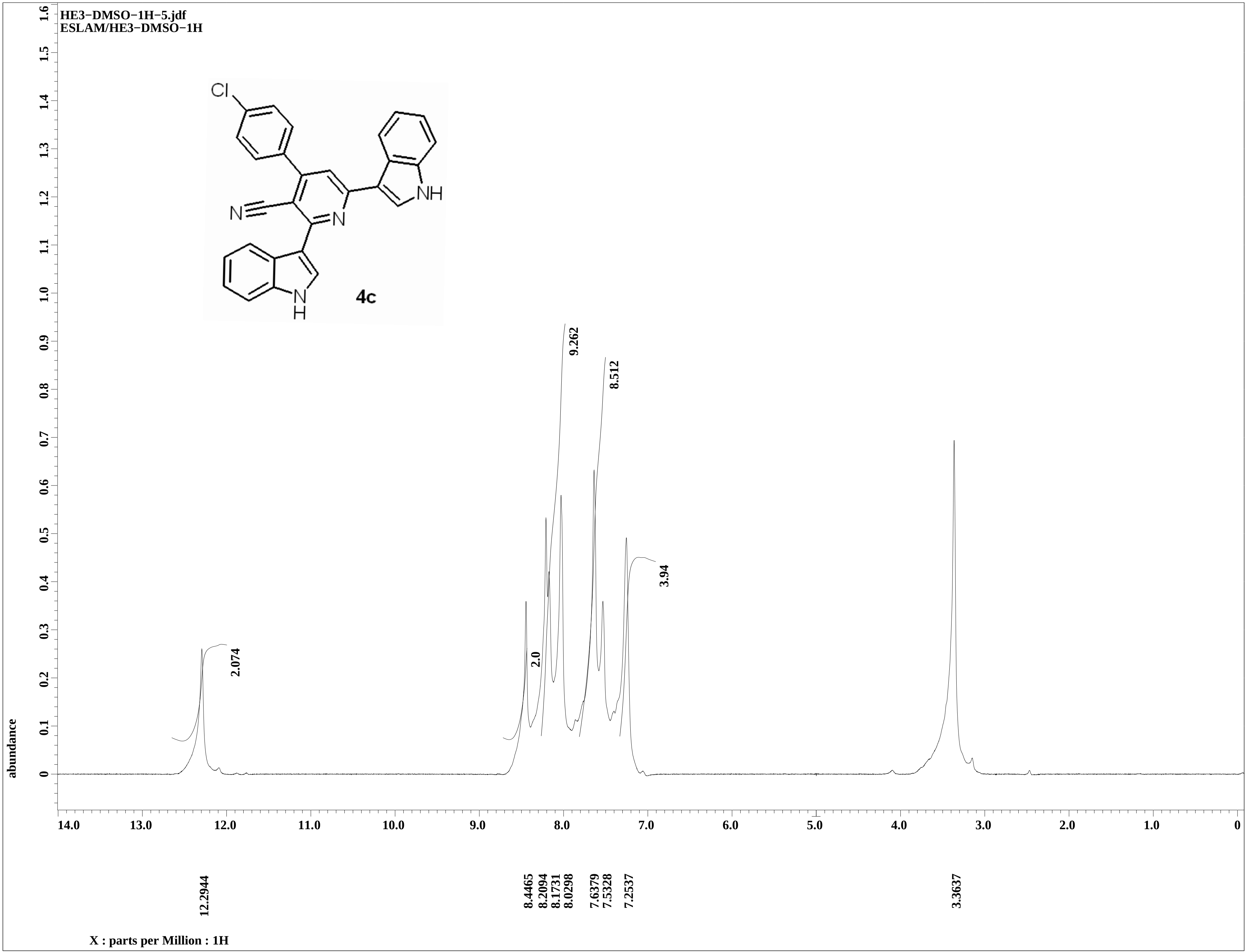
HE3−DMSO−1H−5.jdf
ESLAM/HE3−DMSO−1H
Cl
N
NH
N
H
4c
abundance
2.074
2.0
9.262
8.512
3.94
12.2944
8.4465
8.2094
8.1731
8.0298
7.6379
7.5328
7.2537
3.3637
X : parts per Million : 1H

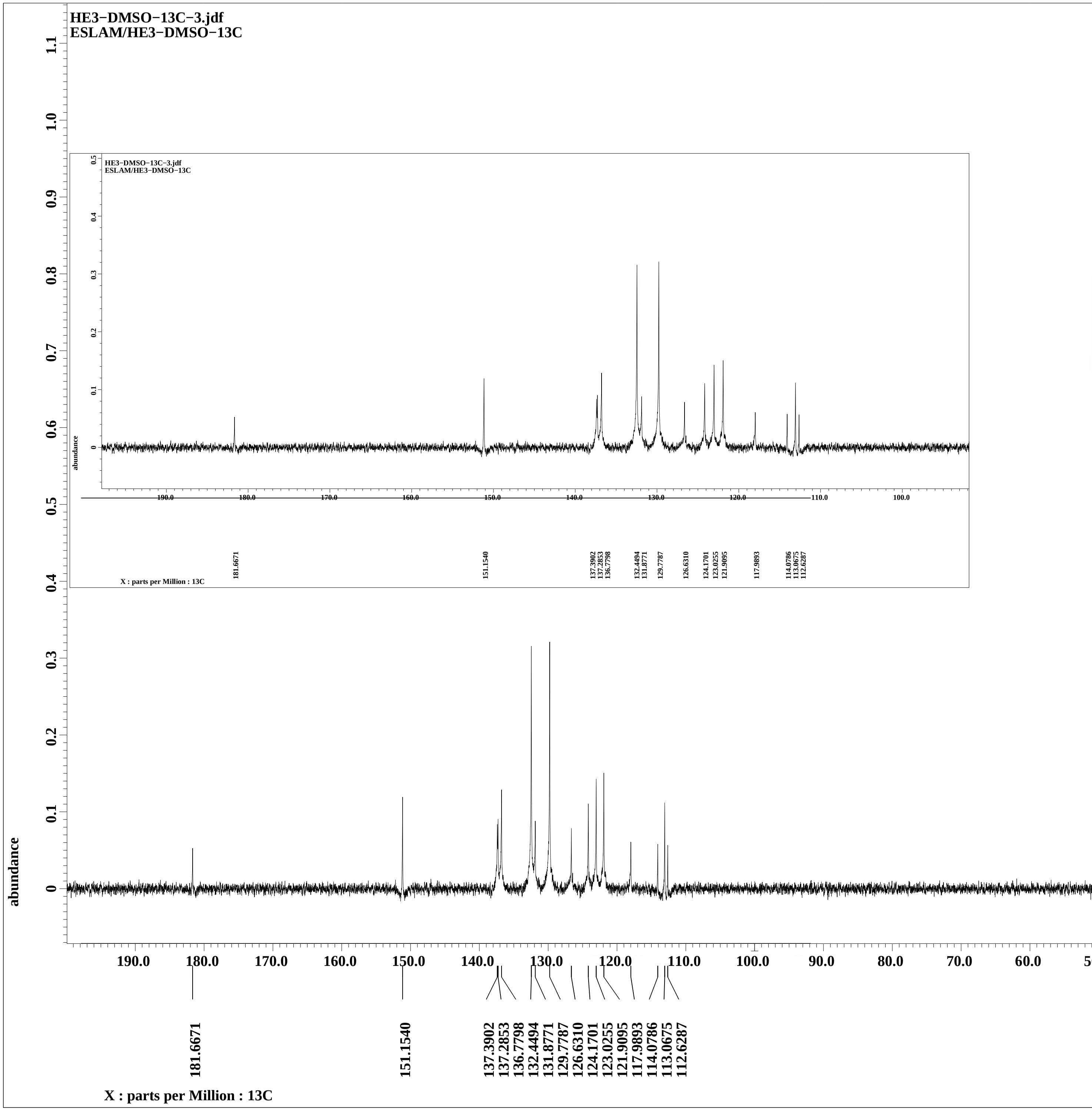
HE3−DMSO−13C−3.jdf
ESLAM/HE3−DMSO−13C
abundance
X : parts per Million : 13C
181.6671
151.1540
137.3902
137.2853
136.7798
132.4494
131.8771
129.7787
126.6310
124.1701
123.0255
121.9095
117.9893
114.0786
113.0675
112.6287
40.2233
40.0516
39.8895

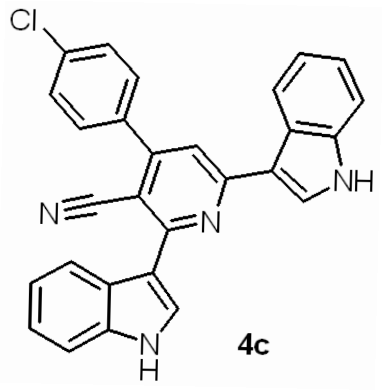
Cl
N
NH
N
H

4c

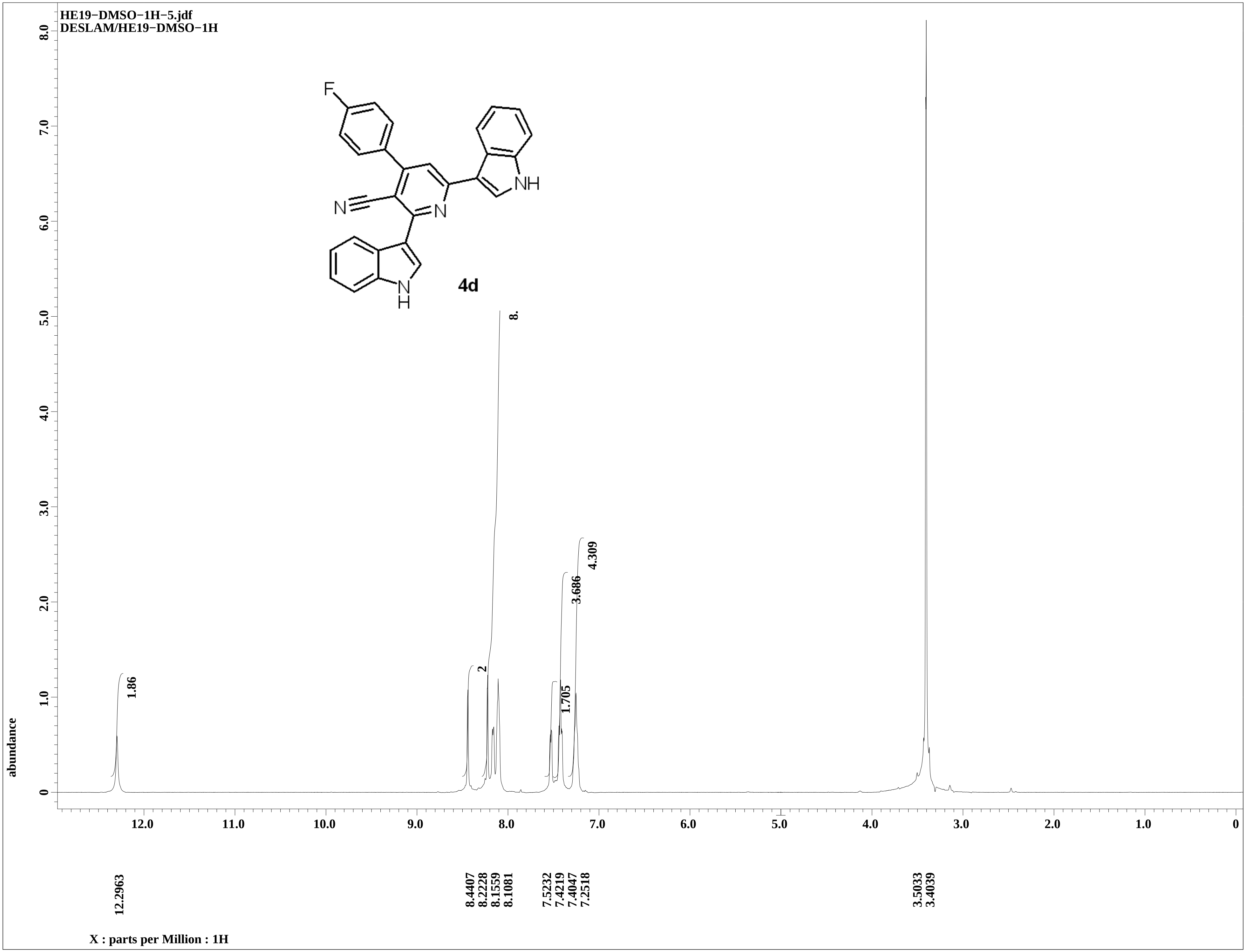
HE19−DMSO−1H−5.jdf
DESLAM/HE19−DMSO−1H
4d
abundance
X : parts per Million : 1H
12.2963
8.4407
8.2228
8.1559
8.1081
7.5232
7.4219
7.4047
7.2518
3.5033
3.4039
1.86
2
8.
1.705
3.686
4.309

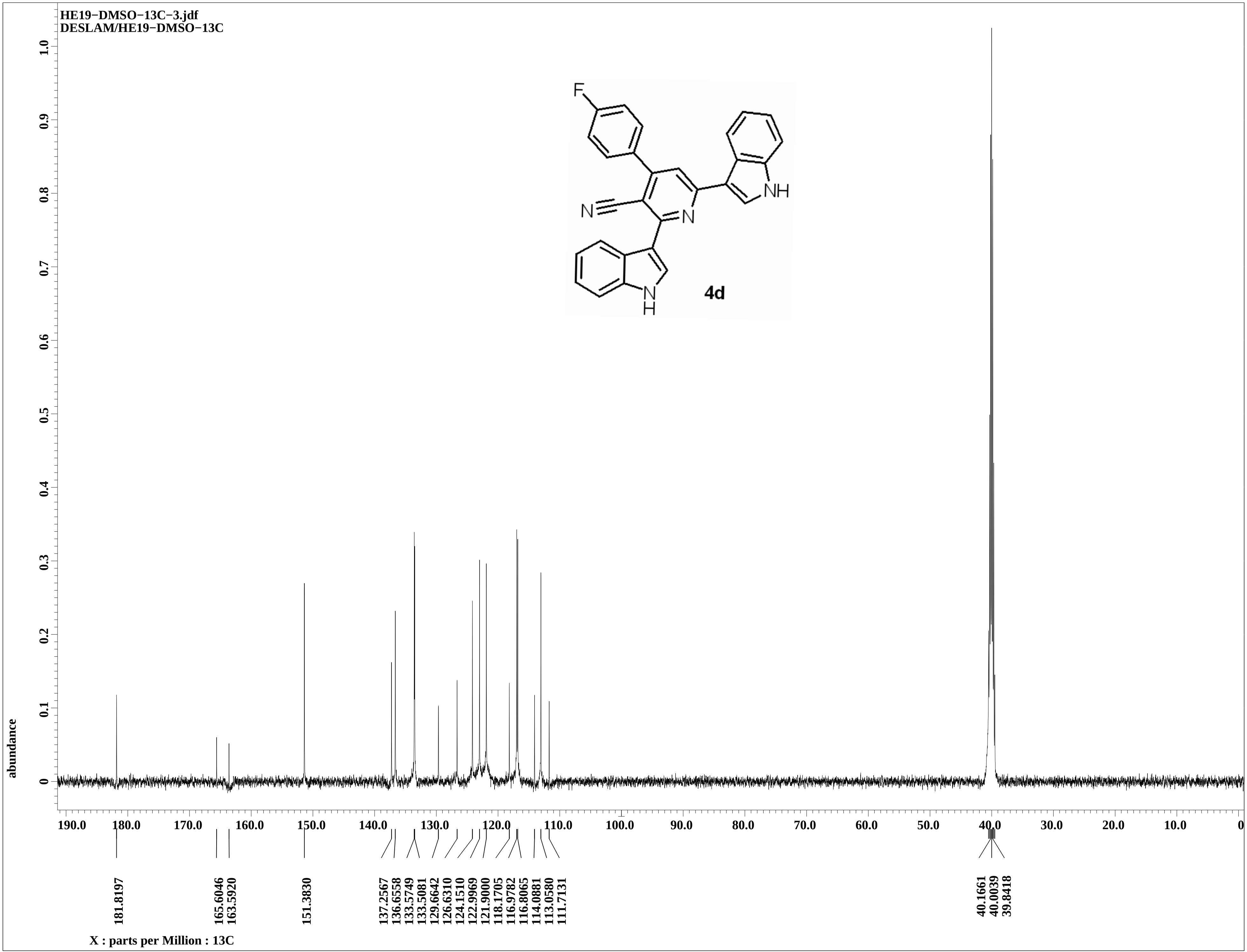
HE19−DMSO−13C−3.jdf
DESLAM/HE19−DMSO−13C
F
N
NH
N
H
4d
abundance
0
0.1
0.2
0.3
0.4
0.5
0.6
0.7
0.8
0.9
1.0
190.0
180.0
170.0
160.0
150.0
140.0
130.0
120.0
110.0
100.0
90.0
80.0
70.0
60.0
50.0
40.0
30.0
20.0
10.0
0
181.8197
165.6046
163.5920
151.3830
137.2567
136.6558
133.5749
133.5081
129.6642
126.6310
124.1510
122.9969
121.9000
118.1705
116.9782
116.8065
114.0881
113.0580
111.7131
40.1661
40.0039
39.8418
X : parts per Million : 13C

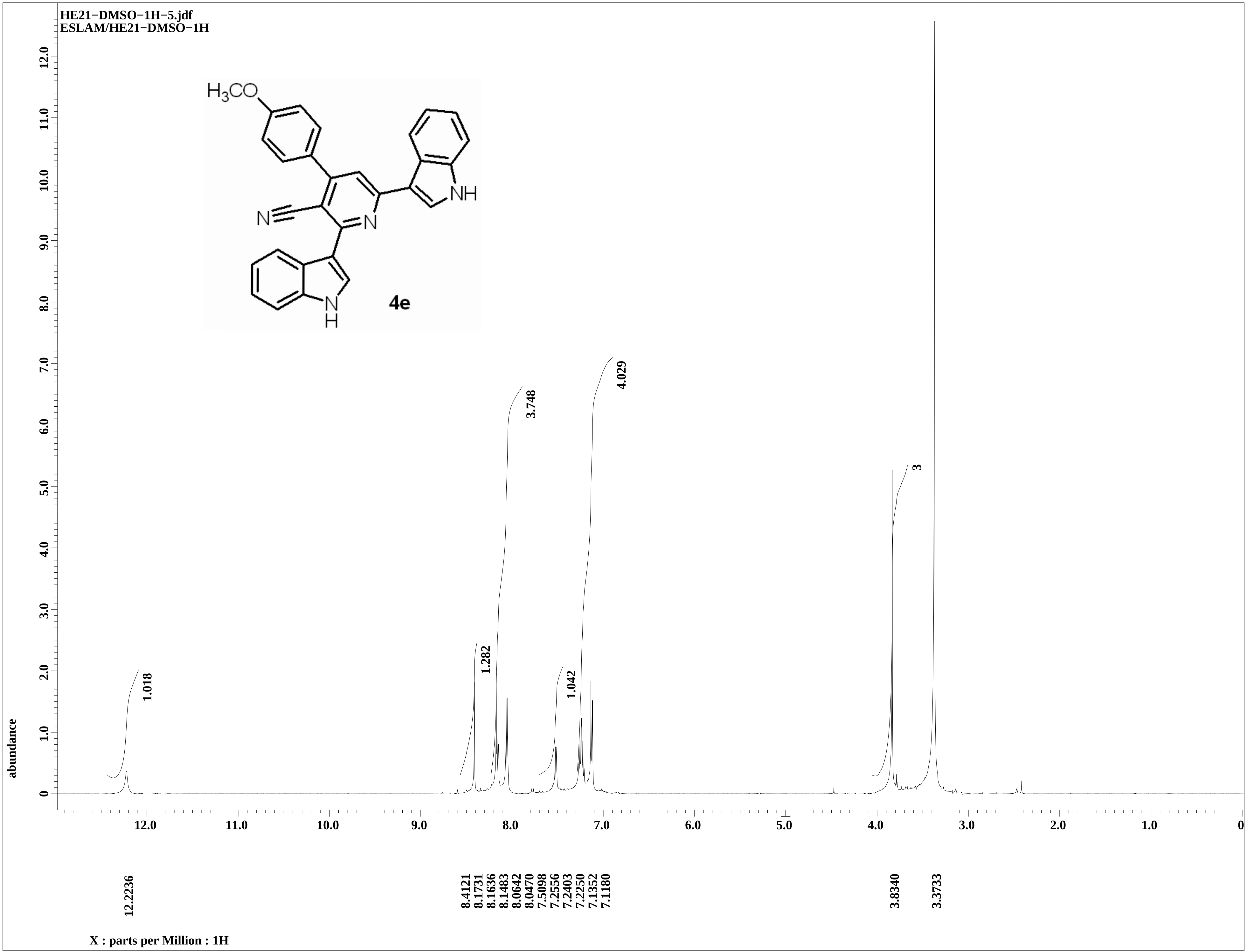
HE21−DMSO−1H−5.jdf
ESLAM/HE21−DMSO−1H
H3CO
N
NH
4e
abundance
1.018
1.282
3.748
1.042
4.029
3
12.2236
8.4121
8.1731
8.1636
8.1483
8.0642
8.0470
7.5098
7.2556
7.2403
7.2250
7.1352
7.1180
3.8340
3.3733
X : parts per Million : 1H

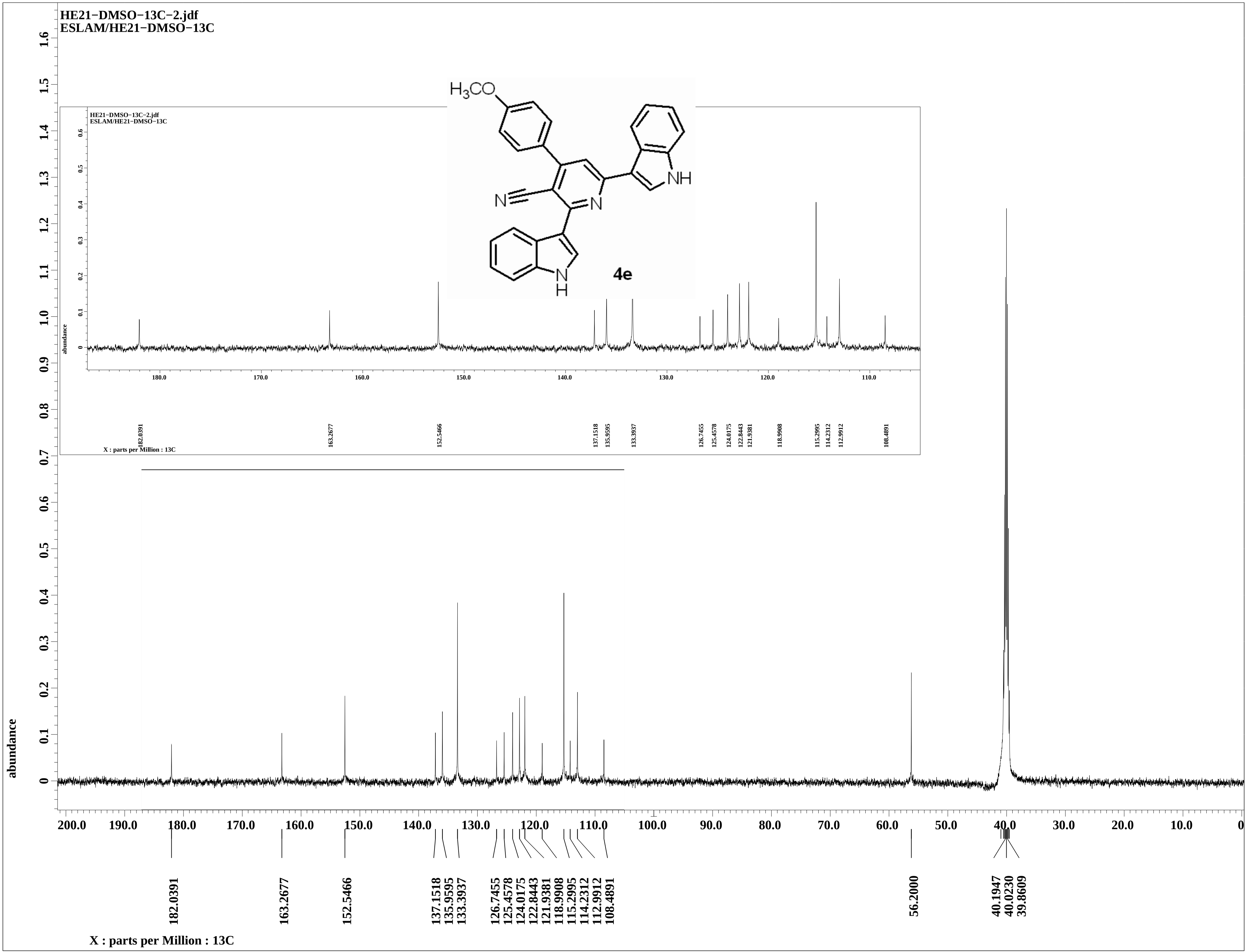
HE21−DMSO−13C−2.jdf
ESLAM/HE21−DMSO−13C
abundance
X : parts per Million : 13C
H3CO
N
NH
N
H
4e
182.0391
163.2677
152.5466
137.1518
135.9595
133.3937
126.7455
125.4578
124.0175
122.8443
121.9381
118.9908
115.2995
114.2312
112.9912
108.4891
56.2000
40.1947
40.0230
39.8609

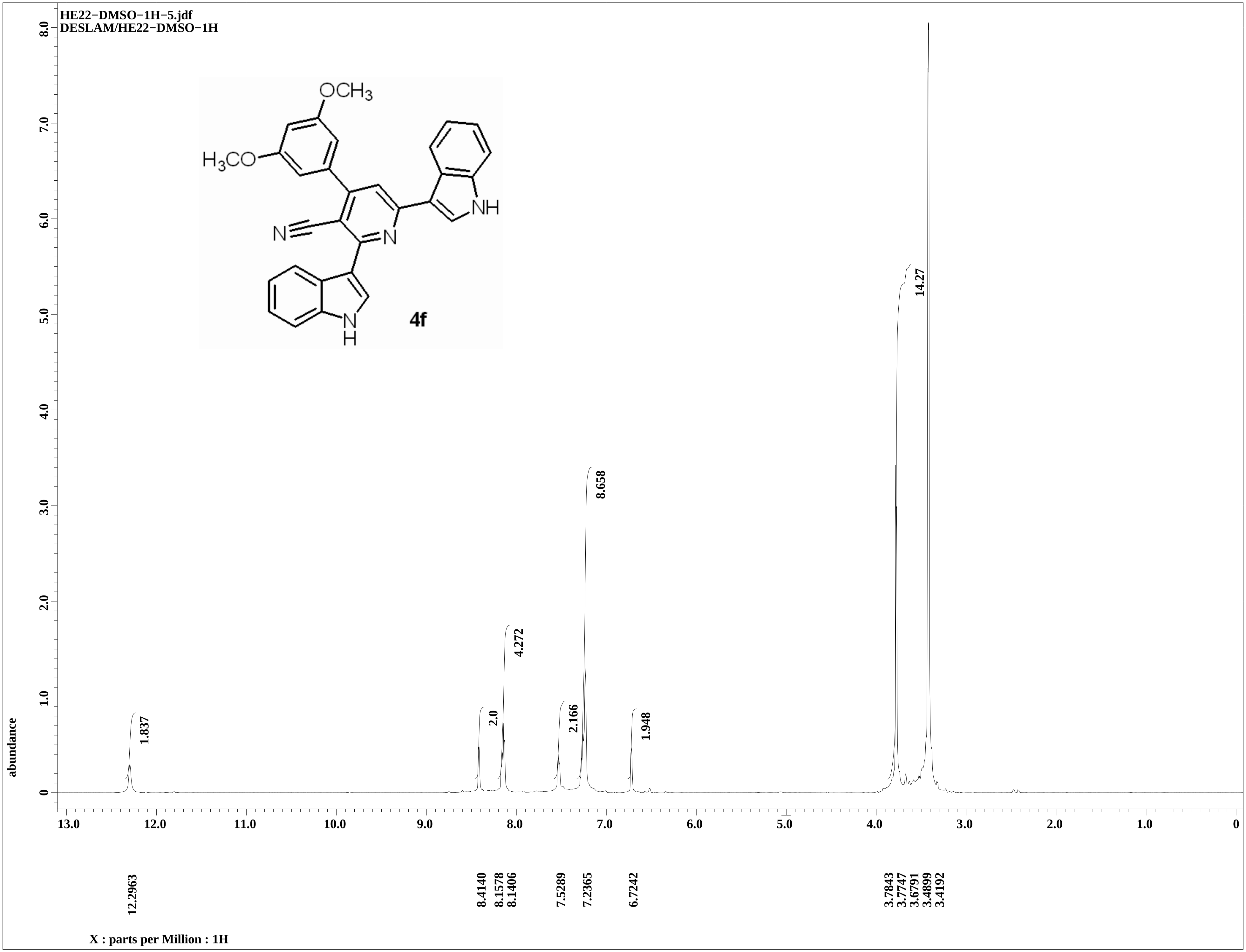
HE22−DMSO−1H−5.jdf
DESLAM/HE22−DMSO−1H
OCH3
H3CO
N
NH
N
H
4f
abundance
1.837
2.0
4.272
2.166
8.658
1.948
14.27
13.0 12.0 11.0 10.0 9.0 8.0 7.0 6.0 5.0 4.0 3.0 2.0 1.0 0
12.2963
8.4140
8.1578
8.1406
7.5289
7.2365
6.7242
3.7843
3.7747
3.6791
3.4899
3.4192
X : parts per Million : 1H

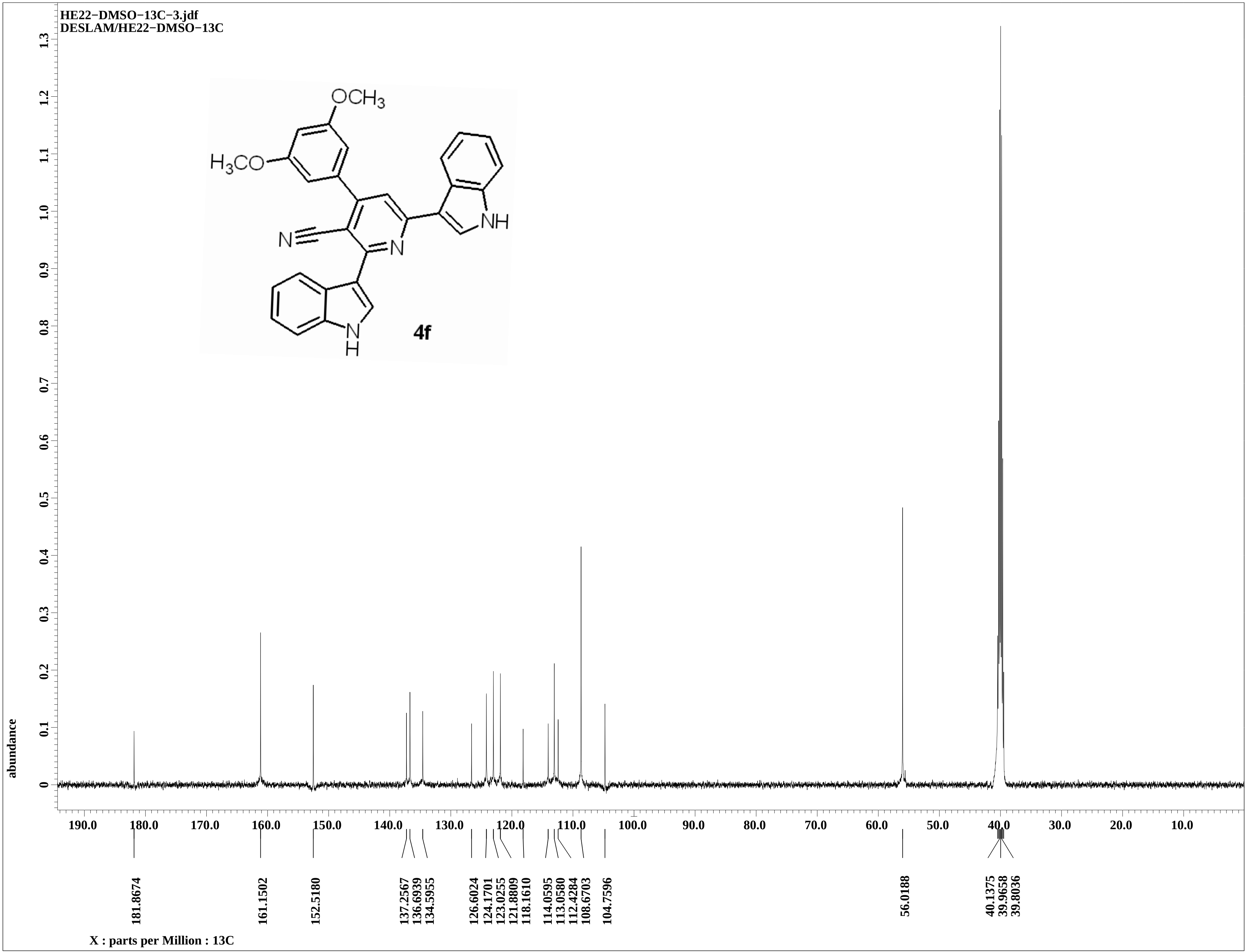
HE22−DMSO−13C−3.jdf
DESLAM/HE22−DMSO−13C
OCH3
H3CO
N
NH
N
H
4f
abundance
0
0.1
0.2
0.3
0.4
0.5
0.6
0.7
0.8
0.9
1.0
1.1
1.2
1.3
190.0
180.0
170.0
160.0
150.0
140.0
130.0
120.0
110.0
100.0
90.0
80.0
70.0
60.0
50.0
40.0
30.0
20.0
10.0
181.8674
161.1502
152.5180
137.2567
136.6939
134.5955
126.6024
124.1701
123.0255
121.8809
118.1610
114.0595
113.0580
112.4284
108.6703
104.7596
56.0188
40.1375
39.9658
39.8036
X : parts per Million : 13C

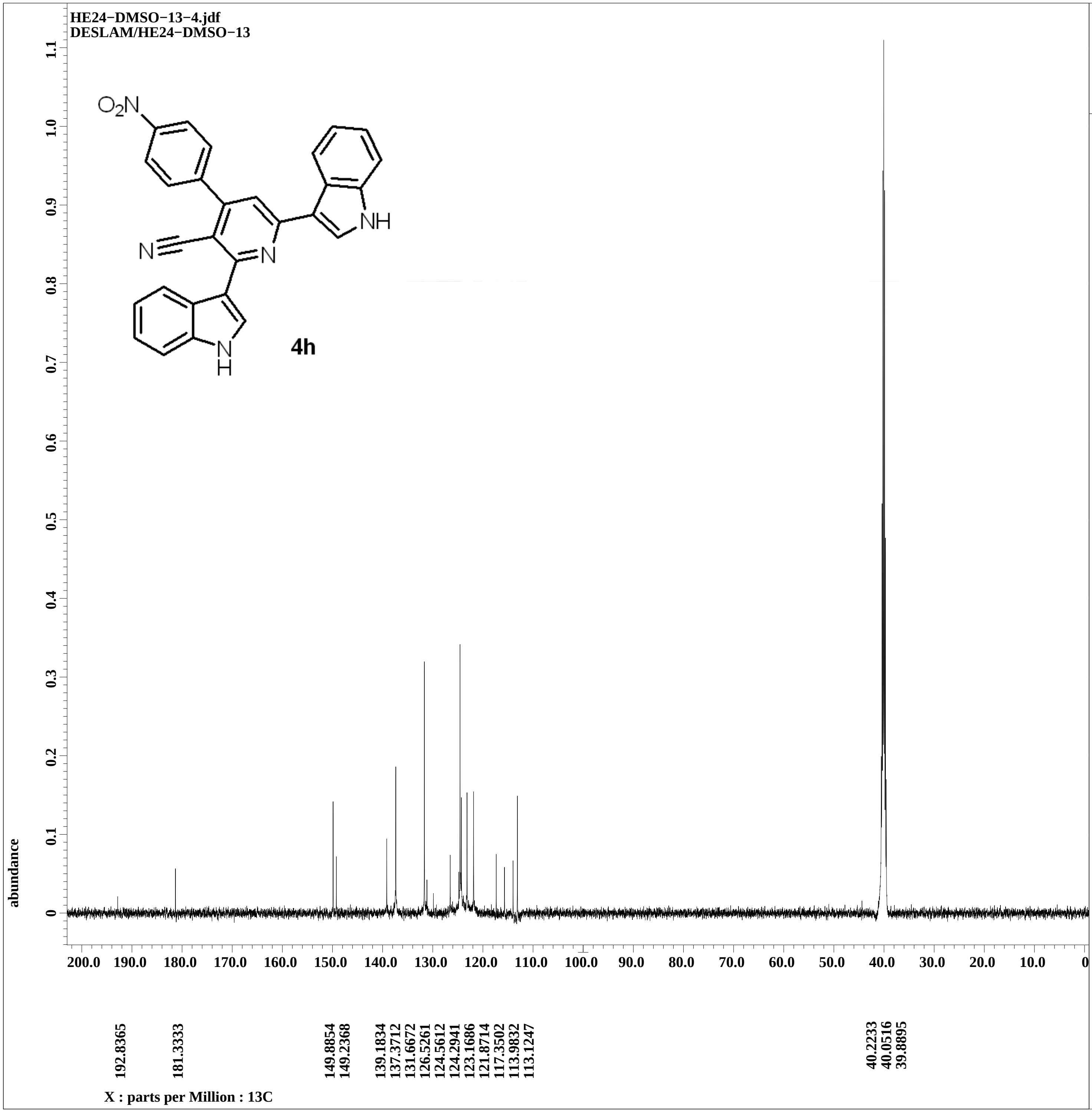

Author	= delta3
Content	= DESLAM/HE24-DMSO
Creation_time	= 14-DEC-2020 18:3
Current_time	= 9-JUN-2021 11:4
Data_format	= 1D REAL
Dim_size	= 26214
Dim_title	= 13C
Dim_units	= [ppm]
Dimensions	= X
Filename	= HE24-DMSO-13-4.j
Machine	= scc
Revision_time	= 9-JUN-2021 11:4
Sample_id	= DESLAM/HE24-DMSO
Site	= ECA500 (Datum BL
Spectrometer	= DELTA2_NMR
Scans	= 694
Mod_return	= 1
Total_scans	= 694
X_points	= 32768
X_prescans	= 4
X_domain	= 13C
X_offset	= 100[ppm]
X_freq	= 125.76529768[MHz
X_sweep	= 39.3081761[kHz]
X_resolution	= 1.19959034[Hz]
Irr_domain	= 1H
Irr_offset	= 5.0[ppm]
Irr_freq	= 500.15991521[MHz
X_acq_duration	= 0.83361792[s]
Digital_filter	= TRUE
Filter_factor	= 8
Af_version	= 1
Delay_of_start	= 1.99999974[s]
Actual_start_time	= 14-DEC-2020 18:3
Acq_delay	= 20.67[us]
Digital_filter_status	= 2P
Clipped	= TRUE
Dc_balanced	= FALSE
X90	= 13[us]
Irr90	= 12[us]
Tri90	= 10[us]
Qua90	= 10[us]
Qui90	= 10[us]
Sex90	= 10[us]
Sep90	= 10[us]
Oct90	= 10[us]
Non90	= 10[us]
Dec90	= 10[us]
X90_hi	= 0.118[ms]
Irr90_hi	= 92[us]
Tri90_hi	= 10[us]
Qua90_hi	= 10[us]
Qui90_hi	= 10[us]
Sex90_hi	= 10[us]
Sep90_hi	= 10[us]
Oct90_hi	= 10[us]
Non90_hi	= 10[us]
Dec90_hi	= 10[us]
X90_lo	= 0.118[ms]
Irr90_lo	= 92[us]
Tri90_lo	= 10[us]
Qua90_lo	= 10[us]
Qui90_lo	= 10[us]
Sex90_lo	= 10[us]
Sep90_lo	= 10[us]
Oct90_lo	= 10[us]
Non90_lo	= 10[us]
Dec90_lo	= 10[us]
X90_spin	= 1[us]
Irr90_spin	= 38[us]

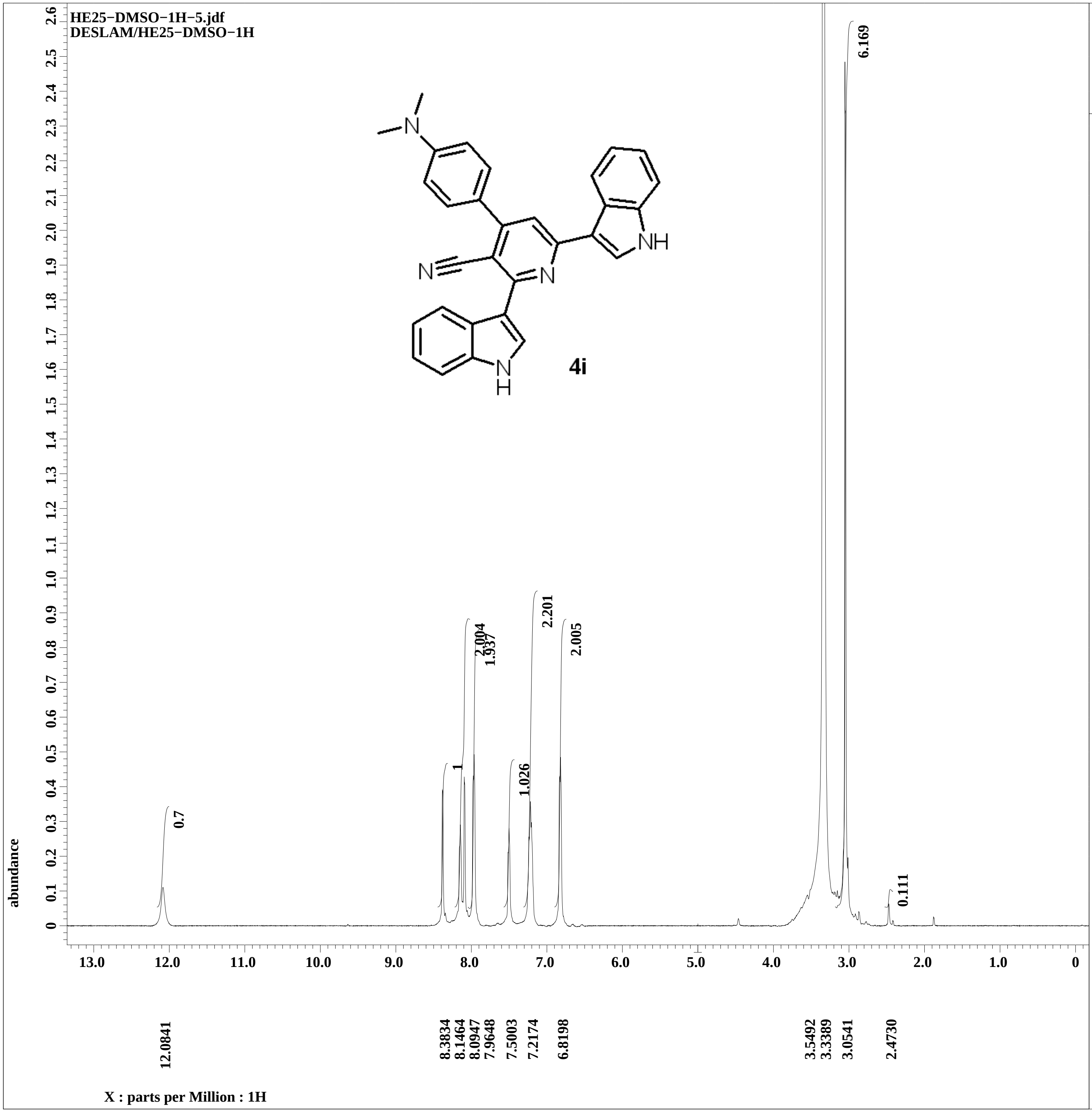

Author	=	delta3
Content	=	DESLAM/HE25-DMSO
Creation_time	=	15-DEC-2020 19:2
Current_time	=	9-JUN-2021 11:4
Data_format	=	1D REAL
Dim_size	=	13107
Dim_title	=	1H
Dim_units	=	[ppm]
Dimensions	=	X
Filename	=	HE25-DMSO-1H-5.j
Machine	=	scc
Revision_time	=	9-JUN-2021 11:4
Sample_id	=	DESLAM/HE25-DMSO
Site	=	ECA500 (Datum BL
Spectrometer	=	DELTA2_NMR
Scans	=	16
Mod_return	=	1
Total_scans	=	16
X_points	=	16384
X_prescans	=	1
X_domain	=	1H
X_offset	=	5.0[ppm]
X_freq	=	500.15991521[MHz
X_sweep	=	15.6641604[kHz]
X_resolution	=	0.95606448[Hz]
Irr_domain	=	1H
Irr_offset	=	5.0[ppm]
Irr_freq	=	500.15991521[MHz
Tri_domain	=	1H
Tri_offset	=	5.0[ppm]
Tri_freq	=	500.15991521[MHz
X_acq_duration	=	1.04595456[s]
Digital_filter	=	TRUE
Filter_factor	=	8
Af_version	=	1
Delay_of_start	=	1.99999974[s]
Actual_start_time	=	15-DEC-2020 19:2
Acq_delay	=	7.94[us]
Digital_filter_status	=	2P
Clipped	=	FALSE
Dc_balanced	=	FALSE
X90	=	12[us]
Irr90	=	12[us]
Tri90	=	10[us]
Qua90	=	10[us]
Qui90	=	10[us]
Sex90	=	10[us]
Sep90	=	10[us]
Oct90	=	10[us]
Non90	=	10[us]
Dec90	=	10[us]
X90_hi	=	92[us]
Irr90_hi	=	92[us]
Tri90_hi	=	10[us]
Qua90_hi	=	10[us]
Qui90_hi	=	10[us]
Sex90_hi	=	10[us]
Sep90_hi	=	10[us]
Oct90_hi	=	10[us]
Non90_hi	=	10[us]
Dec90_hi	=	10[us]
X90_lo	=	92[us]
Irr90_lo	=	92[us]
Tri90_lo	=	10[us]
Qua90_lo	=	10[us]
Qui90_lo	=	10[us]
Sex90_lo	=	10[us]
Sep90_lo	=	10[us]
Oct90_lo	=	10[us]
Non90_lo	=	10[us]

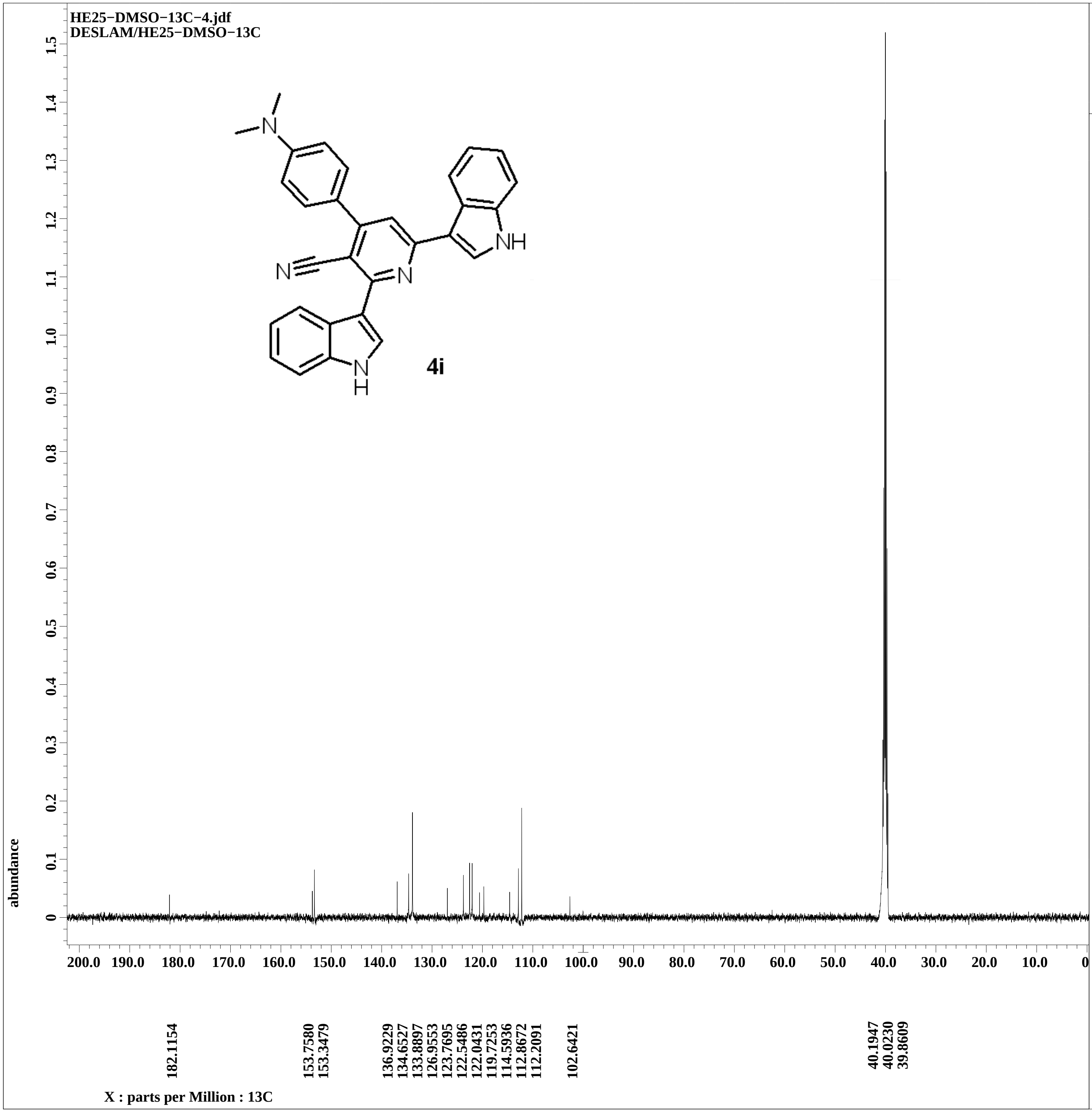

Parameter	Value
Author	= delta3
Content	= DESLAM/HE25-DMSO
Creation_time	= 16-DEC-2020 17:2
Current_time	= 9-JUN-2021 11:3
Data_format	= 1D REAL
Dim_size	= 26214
Dim_title	= 13C
Dim_units	= [ppm]
Dimensions	= X
Filename	= HE25-DMSO-13C-4.
Machine	= scc
Revision_time	= 9-JUN-2021 11:3
Sample_id	= DESLAM/HE25-DMSO
Site	= ECA500 (Datum BL
Spectrometer	= DELTA2_NMR
Scans	= 843
Mod_return	= 1
Total_scans	= 843
X_points	= 32768
X_prescans	= 4
X_domain	= 13C
X_offset	= 100[ppm]
X_freq	= 125.76529768[MHz
X_sweep	= 39.3081761[kHz]
X_resolution	= 1.19959034[Hz]
Irr_domain	= 1H
Irr_offset	= 5.0[ppm]
Irr_freq	= 500.15991521[MHz
X_acq_duration	= 0.83361792[s]
Digital_filter	= TRUE
Filter_factor	= 8
Af_version	= 1
Delay_of_start	= 1.99999974[s]
Actual_start_time	= 16-DEC-2020 17:2
Acq_delay	= 20.67[us]
Digital_filter_status	= 2P
Clipped	= FALSE
Dc_balanced	= FALSE
X90	= 13[us]
Irr90	= 12[us]
Tri90	= 10[us]
Qua90	= 10[us]
Qui90	= 10[us]
Sex90	= 10[us]
Sep90	= 10[us]
Oct90	= 10[us]
Non90	= 10[us]
Dec90	= 10[us]
X90_hi	= 0.118[ms]
Irr90_hi	= 92[us]
Tri90_hi	= 10[us]
Qua90_hi	= 10[us]
Qui90_hi	= 10[us]
Sex90_hi	= 10[us]
Sep90_hi	= 10[us]
Oct90_hi	= 10[us]
Non90_hi	= 10[us]
Dec90_hi	= 10[us]
X90_lo	= 0.118[ms]
Irr90_lo	= 92[us]
Tri90_lo	= 10[us]
Qua90_lo	= 10[us]
Qui90_lo	= 10[us]
Sex90_lo	= 10[us]
Sep90_lo	= 10[us]
Oct90_lo	= 10[us]
Non90_lo	= 10[us]
Dec90_lo	= 10[us]
X90_spin	= 1[us]
Irr90_spin	= 38[us]

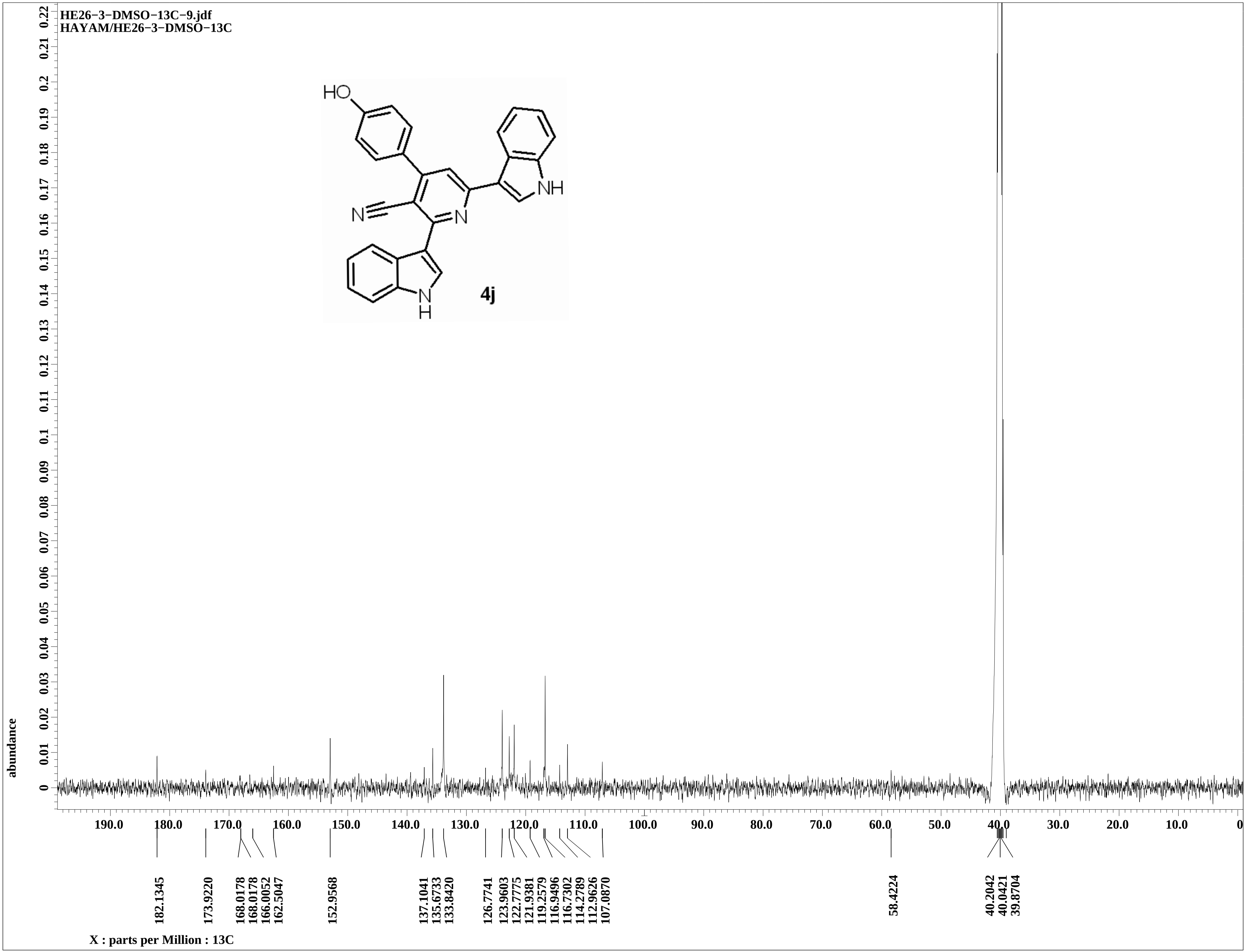
HE26−3−DMSO−13C−9.jdf
HAYAM/HE26−3−DMSO−13C
HO
N
NH
N
H
4j
abundance
182.1345
173.9220
168.0178
168.0178
166.0052
162.5047
152.9568
137.1041
135.6733
133.8420
126.7741
123.9603
122.7775
121.9381
119.2579
116.9496
116.7302
114.2789
112.9626
107.0870
58.4224
40.2042
40.0421
39.8704
X : parts per Million : 13C

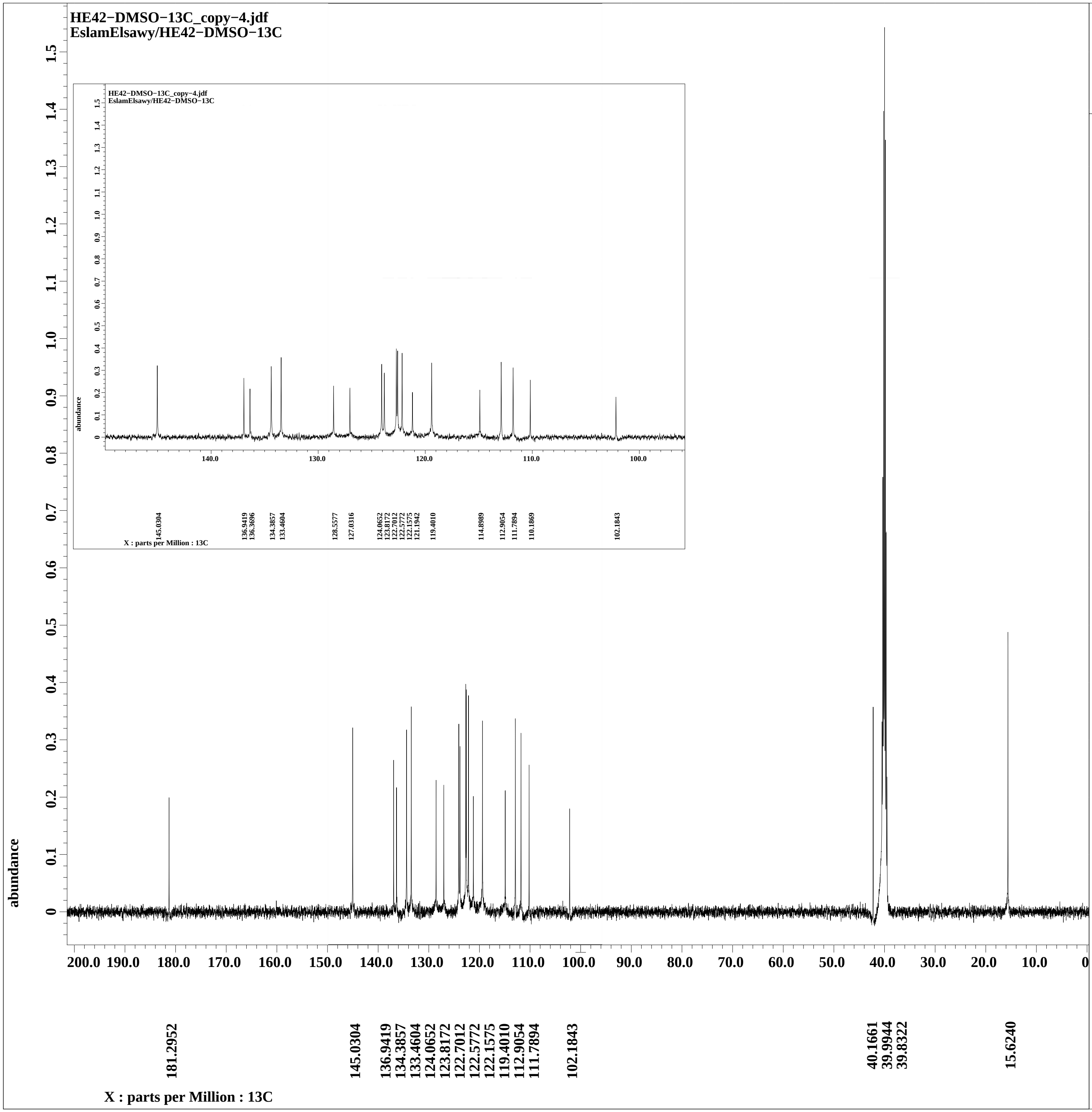

Author = delta3
Content = EslamElsawy/HE42
Creation_time = 22-FEB-2021 19:0
Current_time = 22-FEB-2021 12:0
Data_format = 1D REAL
Dim_size = 26214
Dim_title = 13C
Dim_units = [ppm]
Dime[illegible]
File[illegible] :o
Mach[illegible]
Revi[illegible] 0
Samp[illegible] 2
Site[illegible] L
Spec[illegible]
Scan[illegible]
Mod_[illegible]
Tota[illegible]
X_po[illegible]
X_pr[illegible]
X_do[illegible]
X_of[illegible]
X_fr[illegible] Iz
X_sw[illegible]
X_re[illegible]
Irr_[illegible]
Irr_[illegible]
Irr_[illegible] Iz
X_ac[illegible]

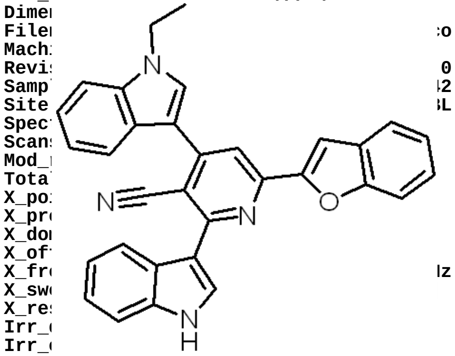

7b

Digital_filter = TRUE
Filter_factor = 8
Af_version = 1
Delay_of_start = 1.99999974[s]
Actual_start_time = 22-FEB-2021 19:0
Acq_delay = 20.67[us]
Digital_filter_status = 2P
Clipped = FALSE
Dc_balanced = FALSE
X90 = 13[us]
Irr90 = 12[us]
Tri90 = 10[us]
Qua90 = 10[us]
Qui90 = 10[us]
Sex90 = 10[us]
Sep90 = 10[us]
Oct90 = 10[us]
Non90 = 10[us]
Dec90 = 10[us]
X90_hi = 0.118[ms]
Irr90_hi = 92[us]
Tri90_hi = 10[us]
Qua90_hi = 10[us]
Qui90_hi = 10[us]
Sex90_hi = 10[us]
Sep90_hi = 10[us]
Oct90_hi = 10[us]
Non90_hi = 10[us]
Dec90_hi = 10[us]
X90_lo = 0.118[ms]
Irr90_lo = 92[us]
Tri90_lo = 10[us]
Qua90_lo = 10[us]
Qui90_lo = 10[us]
Sex90_lo = 10[us]
Sep90_lo = 10[us]
Oct90_lo = 10[us]
Non90_lo = 10[us]
Dec90_lo = 10[us]
X90_spin = 1[us]
Irr90_spin = 38[us]

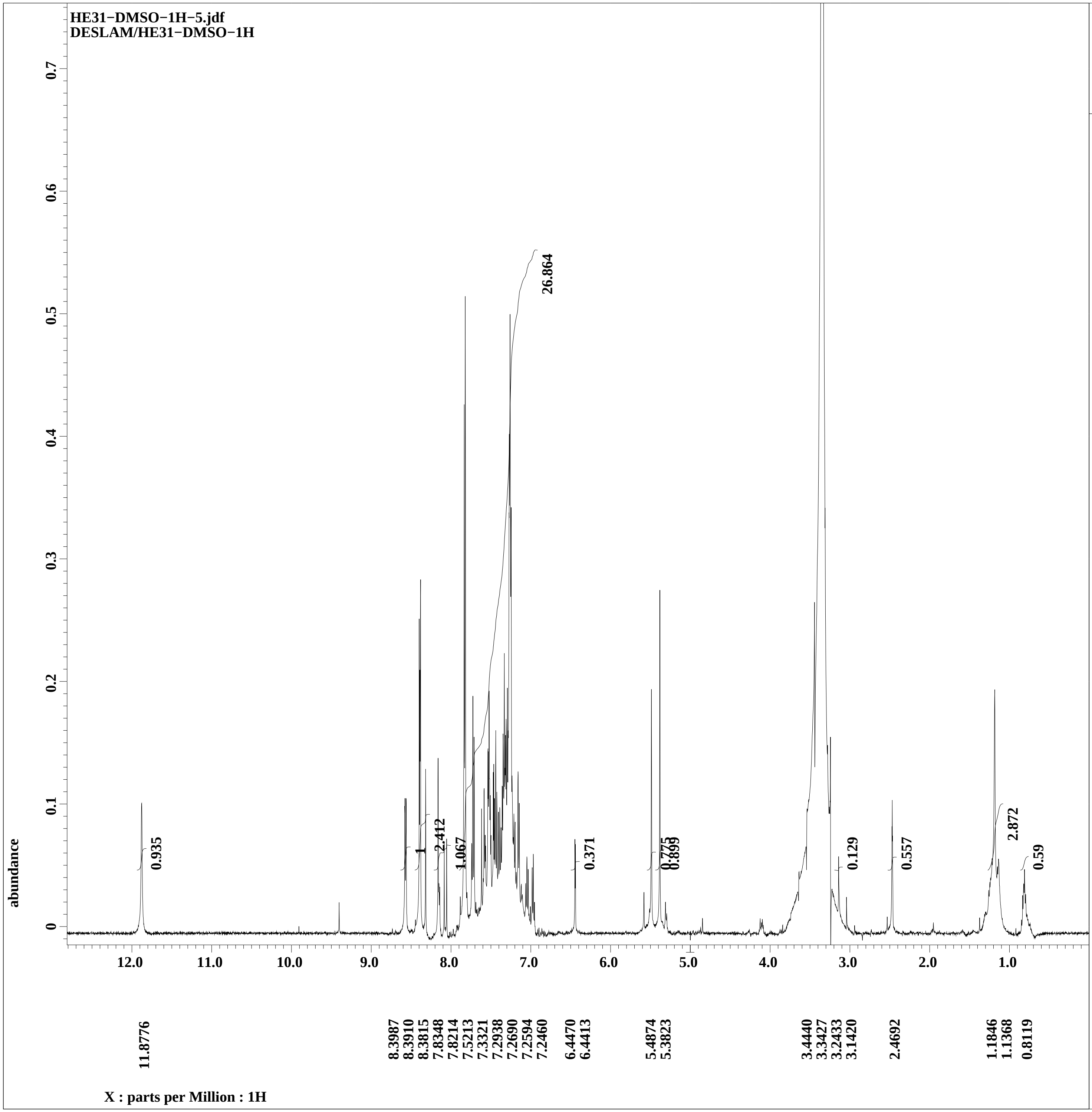

Author	= delta3
Content	= DESLAM/HE31-DMSO
Creation_time	= 24-DEC-2020 18:5
Current_time	= 9-JUN-2021 11:3
Data_format	= 1D REAL
Dim_size	= 13107
Dim_title	= 1H
Dim_units	= [ppm]
Dimensions	= X
Filename	= HE31-DMSO-1H-5.j
Machine	= scc
Revision_time	= 9-JUN-2021 11:3
Sample_id	= DESLAM/HE31-DMSO
Site	= ECA500 (Datum BL
Spectrometer	= DELTA2_NMR
Scans	= 16
Mod_return	= 1
Total_scans	= 16
X_points	= 16384
X_prescans	= 1
X_domain	= 1H
X_offset	= 5.0[ppm]
X_freq	= 500.15991521[MHz
X_sweep	= 15.6641604[kHz]
X_resolution	= 0.95606448[Hz]
Irr_domain	= 1H
Irr_offset	= 5.0[ppm]
Irr_freq	= 500.15991521[MHz
Tri_domain	= 1H
Tri_offset	= 5.0[ppm]
Tri_freq	= 500.15991521[MHz
X_acq_duration	= 1.04595456[s]
Digital_filter	= TRUE
Filter_factor	= 8
Af_version	= 1
Delay_of_start	= 1.99999974[s]
Actual_start_time	= 24-DEC-2020 18:5
Acq_delay	= 7.94[us]
Digital_filter_status	= 2P
Clipped	= FALSE
Dc_balanced	= FALSE
X90	= 12[us]
Irr90	= 12[us]
Tri90	= 10[us]
Qua90	= 10[us]
Qui90	= 10[us]
Sex90	= 10[us]
Sep90	= 10[us]
Oct90	= 10[us]
Non90	= 10[us]
Dec90	= 10[us]
X90_hi	= 92[us]
Irr90_hi	= 92[us]
Tri90_hi	= 10[us]
Qua90_hi	= 10[us]
Qui90_hi	= 10[us]
Sex90_hi	= 10[us]
Sep90_hi	= 10[us]
Oct90_hi	= 10[us]
Non90_hi	= 10[us]
Dec90_hi	= 10[us]
X90_lo	= 92[us]
Irr90_lo	= 92[us]
Tri90_lo	= 10[us]
Qua90_lo	= 10[us]
Qui90_lo	= 10[us]
Sex90_lo	= 10[us]
Sep90_lo	= 10[us]
Oct90_lo	= 10[us]
Non90_lo	= 10[us]

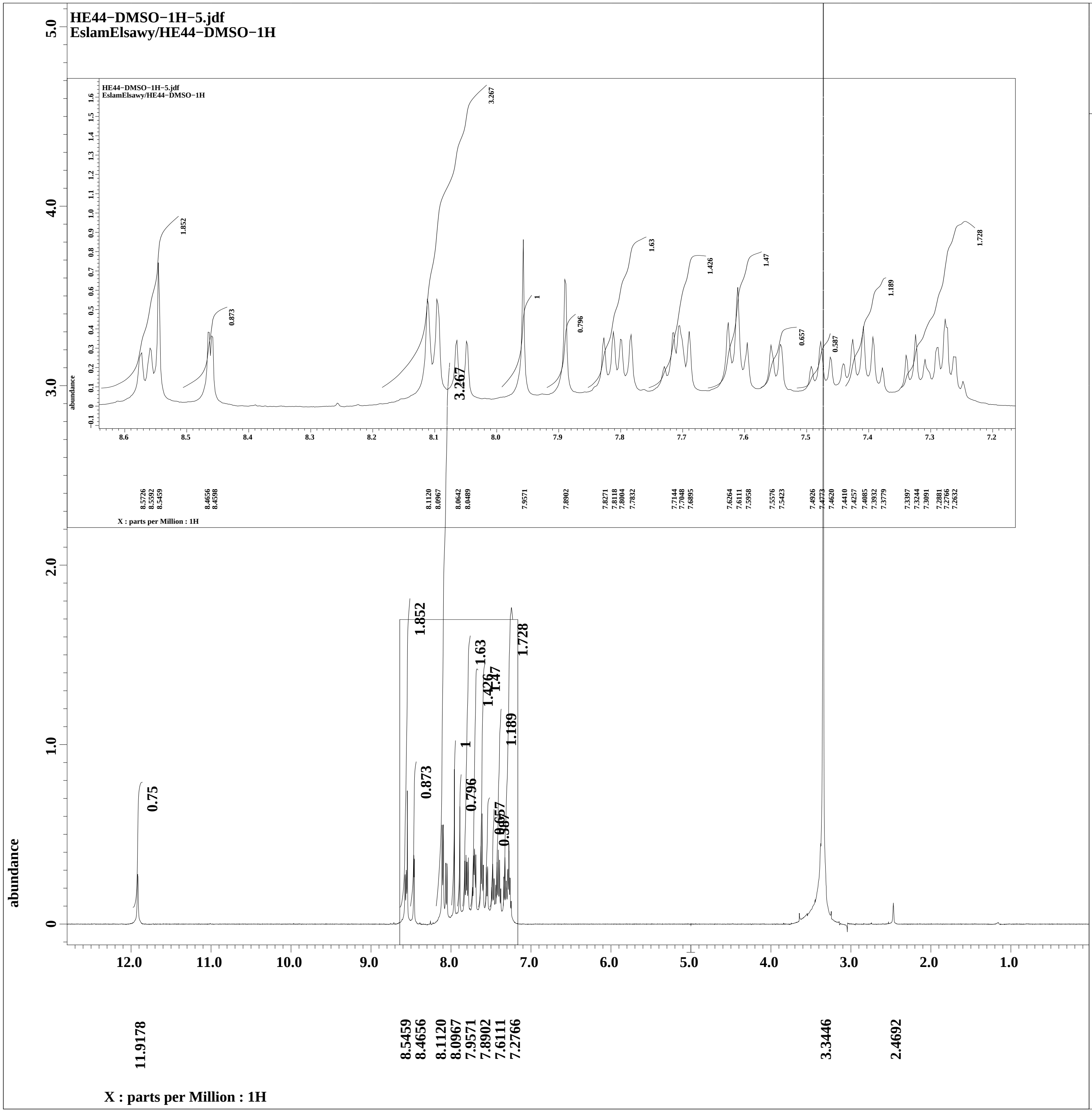

Author	=	delta3
Content	=	EslamElsawy/HE44
Creation_time	=	18-FEB-2021 19:4
Current_time	=	18-FEB-2021 12:4
Data_format	=	1D REAL
Dim_size	=	13107
Dim_title	=	1H
Dim_units	=	[ppm]

SO-1H-5.j
2021 12:4
sawy/HE44
(Datum BL
NMR

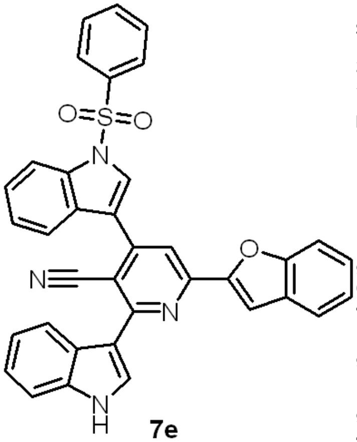

7e

]
91521[MHz
604[kHz]
448[Hz]
]
91521[MHz
]
91521[MHz
456[s]

Digital_filter	=	TRUE
Filter_factor	=	8
Af_version	=	1
Delay_of_start	=	1.99999974[s]
Actual_start_time	=	18-FEB-2021 19:4
Acq_delay	=	7.94[us]
Digital_filter_status	=	2P
Clipped	=	FALSE
Dc_balanced	=	FALSE
X90	=	12[us]
Irr90	=	12[us]
Tri90	=	10[us]
Qua90	=	10[us]
Qui90	=	10[us]
Sex90	=	10[us]
Sep90	=	10[us]
Oct90	=	10[us]
Non90	=	10[us]
Dec90	=	10[us]
X90_hi	=	92[us]
Irr90_hi	=	92[us]
Tri90_hi	=	10[us]
Qua90_hi	=	10[us]
Qui90_hi	=	10[us]
Sex90_hi	=	10[us]
Sep90_hi	=	10[us]
Oct90_hi	=	10[us]
Non90_hi	=	10[us]
Dec90_hi	=	10[us]
X90_lo	=	92[us]
Irr90_lo	=	92[us]
Tri90_lo	=	10[us]
Qua90_lo	=	10[us]
Qui90_lo	=	10[us]
Sex90_lo	=	10[us]
Sep90_lo	=	10[us]
Oct90_lo	=	10[us]
Non90_lo	=	10[us]

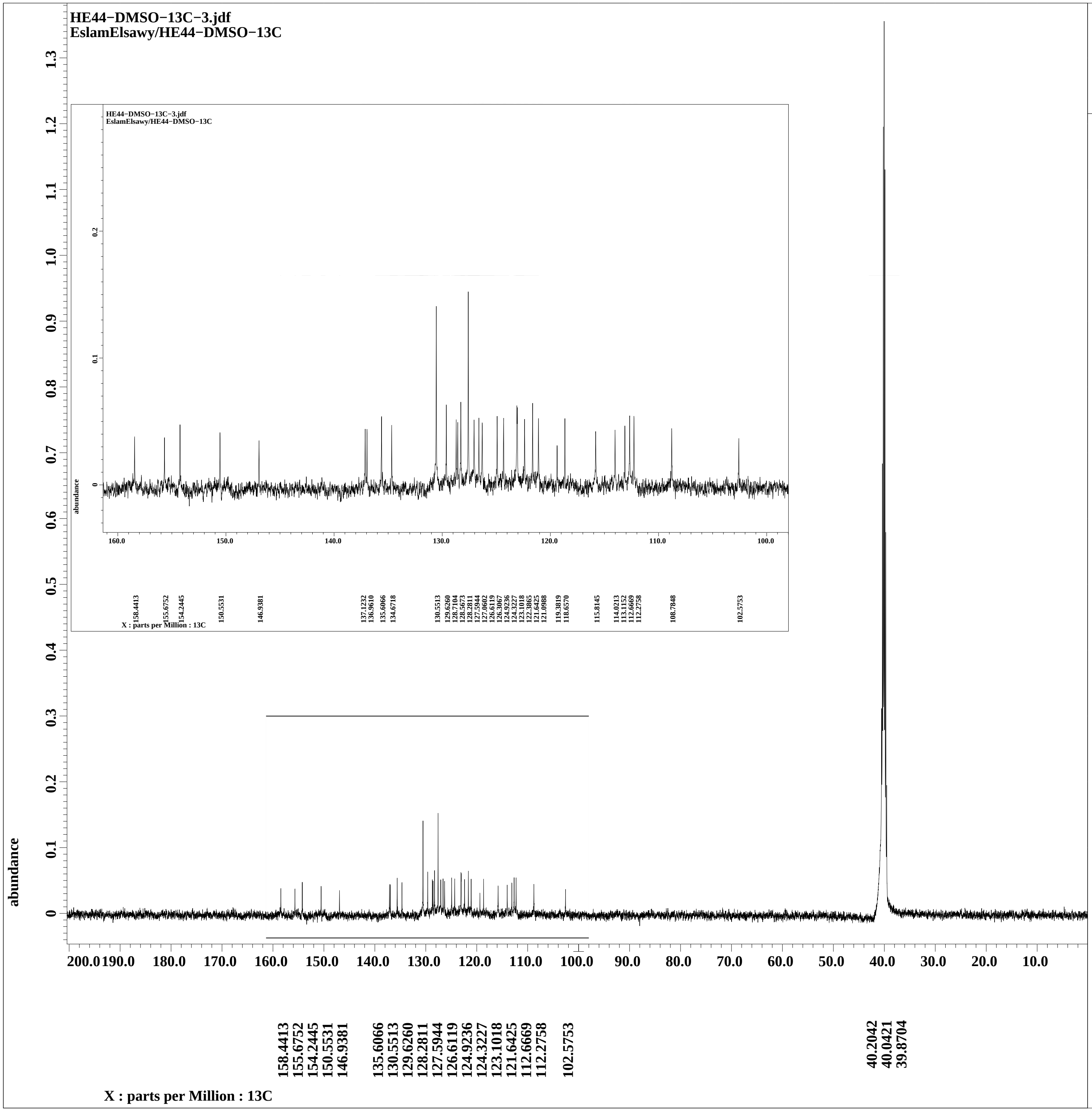

Author = delta3
Content = EslamElsawy/HE44
Creation_time = 18-FEB-2021 20:3
Current_time = 22-FEB-2021 11:5
Data_format = 1D COMPLEX
Dim_size = 26214
Dim_title = 13C
Dim_units = [ppm]

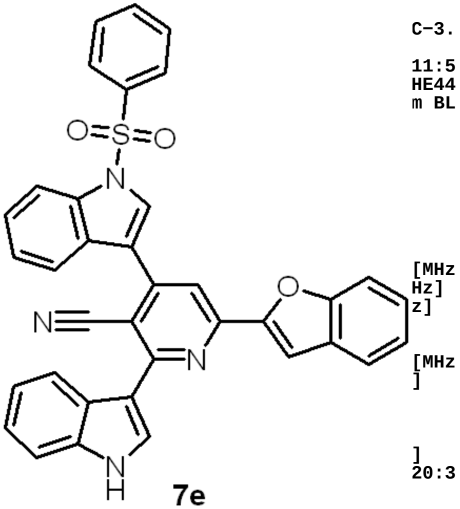

7e

C-3.
11:5
HE44
m BL
[MHz
Hz]
z]
[MHz
]
]
20:3

Clipped = FALSE
Dc_balanced = FALSE
X90 = 13[us]
Irr90 = 12[us]
Tri90 = 10[us]
Qua90 = 10[us]
Qui90 = 10[us]
Sex90 = 10[us]
Sep90 = 10[us]
Oct90 = 10[us]
Non90 = 10[us]
Dec90 = 10[us]
X90_hi = 0.118[ms]
Irr90_hi = 92[us]
Tri90_hi = 10[us]
Qua90_hi = 10[us]
Qui90_hi = 10[us]
Sex90_hi = 10[us]
Sep90_hi = 10[us]
Oct90_hi = 10[us]
Non90_hi = 10[us]
Dec90_hi = 10[us]
X90_lo = 0.118[ms]
Irr90_lo = 92[us]
Tri90_lo = 10[us]
Qua90_lo = 10[us]
Qui90_lo = 10[us]
Sex90_lo = 10[us]
Sep90_lo = 10[us]
Oct90_lo = 10[us]
Non90_lo = 10[us]
Dec90_lo = 10[us]
X90_spin = 1[us]
Irr90_spin = 38[us]

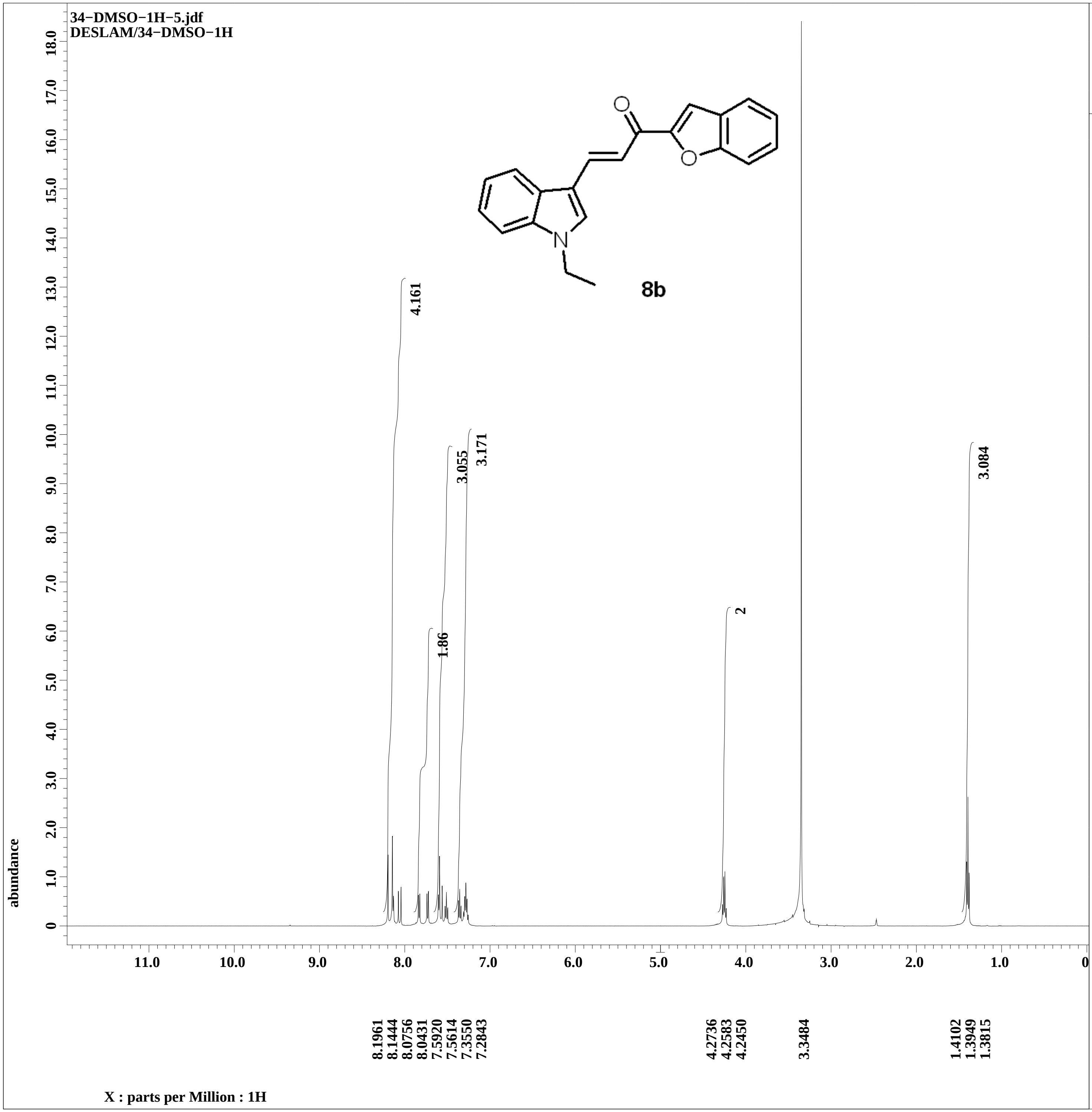

Author	=	delta3
Content	=	DESLAM/34-DMSO-1
Creation_time	=	16-DEC-2020 18:5
Current_time	=	9-JUN-2021 11:2
Data_format	=	1D REAL
Dim_size	=	13107
Dim_title	=	1H
Dim_units	=	[ppm]
Dimensions	=	X
Filename	=	34-DMSO-1H-5.jdf
Machine	=	scc
Revision_time	=	9-JUN-2021 11:2
Sample_id	=	DESLAM/34-DMSO-1
Site	=	ECA500 (Datum BL
Spectrometer	=	DELTA2_NMR
Scans	=	16
Mod_return	=	1
Total_scans	=	16
X_points	=	16384
X_prescans	=	1
X_domain	=	1H
X_offset	=	5.0[ppm]
X_freq	=	500.15991521[MHz
X_sweep	=	15.6641604[kHz]
X_resolution	=	0.95606448[Hz]
Irr_domain	=	1H
Irr_offset	=	5.0[ppm]
Irr_freq	=	500.15991521[MHz
Tri_domain	=	1H
Tri_offset	=	5.0[ppm]
Tri_freq	=	500.15991521[MHz
X_acq_duration	=	1.04595456[s]
Digital_filter	=	TRUE
Filter_factor	=	8
Af_version	=	1
Delay_of_start	=	1.99999974[s]
Actual_start_time	=	16-DEC-2020 18:5
Acq_delay	=	7.94[us]
Digital_filter_status	=	2P
Clipped	=	FALSE
Dc_balanced	=	FALSE
X90	=	12[us]
Irr90	=	12[us]
Tri90	=	10[us]
Qua90	=	10[us]
Qui90	=	10[us]
Sex90	=	10[us]
Sep90	=	10[us]
Oct90	=	10[us]
Non90	=	10[us]
Dec90	=	10[us]
X90_hi	=	92[us]
Irr90_hi	=	92[us]
Tri90_hi	=	10[us]
Qua90_hi	=	10[us]
Qui90_hi	=	10[us]
Sex90_hi	=	10[us]
Sep90_hi	=	10[us]
Oct90_hi	=	10[us]
Non90_hi	=	10[us]
Dec90_hi	=	10[us]
X90_lo	=	92[us]
Irr90_lo	=	92[us]
Tri90_lo	=	10[us]
Qua90_lo	=	10[us]
Qui90_lo	=	10[us]
Sex90_lo	=	10[us]
Sep90_lo	=	10[us]
Oct90_lo	=	10[us]
Non90_lo	=	10[us]

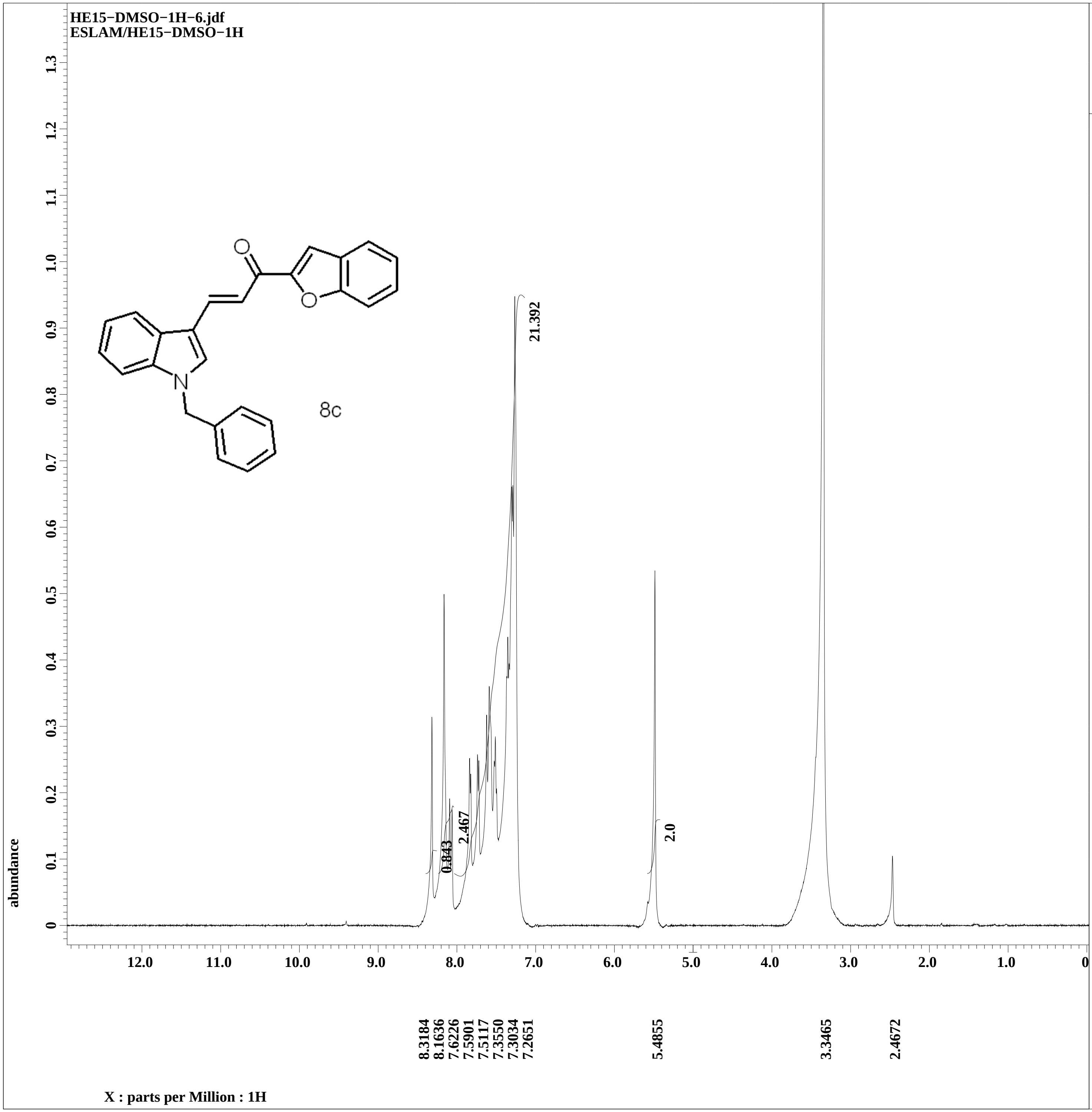

Author	= delta3
Content	= ESLAM/HE15-DMSO-
Creation_time	= 8-JUN-2021 18:2
Current_time	= 8-JUN-2021 11:1
Data_format	= 1D REAL
Dim_size	= 13107
Dim_title	= 1H
Dim_units	= [ppm]
Dimensions	= X
Filename	= HE15-DMSO-1H-6.j
Machine	= scc
Revision_time	= 8-JUN-2021 11:1
Sample_id	= ESLAM/HE15-DMSO-
Site	= ECA500 (Datum BL
Spectrometer	= DELTA2_NMR
Scans	= 16
Mod_return	= 1
Total_scans	= 16
X_points	= 16384
X_prescans	= 1
X_domain	= 1H
X_offset	= 5.0[ppm]
X_freq	= 500.15991521[MHz
X_sweep	= 15.6641604[kHz]
X_resolution	= 0.95606448[Hz]
Irr_domain	= 1H
Irr_offset	= 5.0[ppm]
Irr_freq	= 500.15991521[MHz
Tri_domain	= 1H
Tri_offset	= 5.0[ppm]
Tri_freq	= 500.15991521[MHz
X_acq_duration	= 1.04595456[s]
Digital_filter	= TRUE
Filter_factor	= 8
Af_version	= 1
Delay_of_start	= 1.99999974[s]
Actual_start_time	= 8-JUN-2021 18:1
Acq_delay	= 7.94[us]
Digital_filter_status	= 2P
Clipped	= FALSE
Dc_balanced	= FALSE
X90	= 12[us]
Irr90	= 12[us]
Tri90	= 10[us]
Qua90	= 10[us]
Qui90	= 10[us]
Sex90	= 10[us]
Sep90	= 10[us]
Oct90	= 10[us]
Non90	= 10[us]
Dec90	= 10[us]
X90_hi	= 92[us]
Irr90_hi	= 92[us]
Tri90_hi	= 10[us]
Qua90_hi	= 10[us]
Qui90_hi	= 10[us]
Sex90_hi	= 10[us]
Sep90_hi	= 10[us]
Oct90_hi	= 10[us]
Non90_hi	= 10[us]
Dec90_hi	= 10[us]
X90_lo	= 92[us]
Irr90_lo	= 92[us]
Tri90_lo	= 10[us]
Qua90_lo	= 10[us]
Qui90_lo	= 10[us]
Sex90_lo	= 10[us]
Sep90_lo	= 10[us]
Oct90_lo	= 10[us]
Non90_lo	= 10[us]

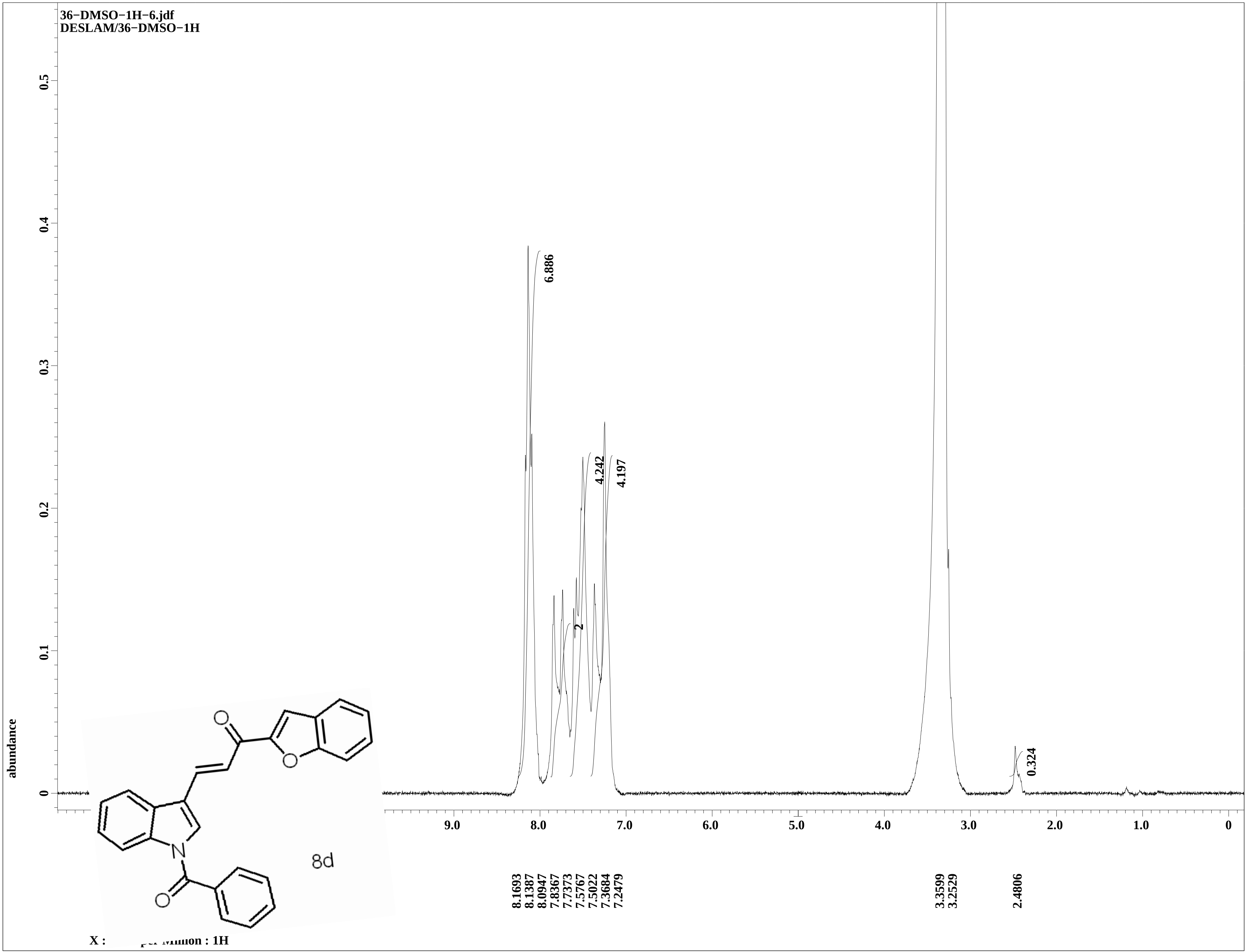
36−DMSO−1H−6.jdf
DESLAM/36−DMSO−1H
abundance
0
0.1
0.2
0.3
0.4
0.5
6.886
4.242
4.197
2
0.324
9.0
8.0
7.0
6.0
5.0
4.0
3.0
2.0
1.0
0
8.1693
8.1387
8.0947
7.8367
7.7373
7.5767
7.5022
7.3684
7.2479
3.3599
3.2529
2.4806
8d
X : parts per Million : 1H
